# Identification of shared and unique gene families associated with oral clefts

**DOI:** 10.1038/ijos.2016.56

**Published:** 2017-01-20

**Authors:** Noriko Funato, Masataka Nakamura

**Affiliations:** 1 Department of Signal Gene Regulation, Research Center for Medical and Dental Sciences, Tokyo Medical and Dental University, 1-5-45 Yushima, Bunkyo-ku, Tokyo, Japan

**Keywords:** cleft palate, epidemiology, gene ontology, mutations, soft palate, syndrome

## Abstract

Oral clefts, the most frequent congenital birth defects in humans, are multifactorial disorders caused by genetic and environmental factors. Epidemiological studies point to different etiologies underlying the oral cleft phenotypes, cleft lip (CL), CL and/or palate (CL/P) and cleft palate (CP). More than 350 genes have syndromic and/or nonsyndromic oral cleft associations in humans. Although genes related to genetic disorders associated with oral cleft phenotypes are known, a gap between detecting these associations and interpretation of their biological importance has remained. Here, using a gene ontology analysis approach, we grouped these candidate genes on the basis of different functional categories to gain insight into the genetic etiology of oral clefts. We identified different genetic profiles and found correlations between the functions of gene products and oral cleft phenotypes. Our results indicate inherent differences in the genetic etiologies that underlie oral cleft phenotypes and support epidemiological evidence that genes associated with CL/P are both developmentally and genetically different from CP only, incomplete CP, and submucous CP. The epidemiological differences among cleft phenotypes may reflect differences in the underlying genetic causes. Understanding the different causative etiologies of oral clefts is important as it may lead to improvements in diagnosis, counseling, and prevention.

## Introduction

Oral clefts are common multifactorial birth defects presenting with a wide range of abnormalities in the upper lip, the primary palate, and the secondary palate, and include cleft lip (CL), cleft palate (CP), CL and/or palate (CL/P), incomplete CP, and submucous CP.^1,2^ Because the secondary palate consists of both a bone-lined hard palate and a bone-free soft palate, incomplete CP includes hard-palate cleft, soft-palate cleft, and bifid uvula. The mildest forms of CP are defects of the soft palate only (soft-palate cleft) or the uvula only (bifid uvula). Oral clefts may be nonsyndromic or manifest as a clinical phenotype within syndromes. They can be caused by different etiological factors such as single gene mutations, chromosomal aberrations, and specific environmental agents as well as by interactions between genetic and environmental influences.^3, 4^ Concordance rates for CL, CL/P, and CP are higher in monozygotic twins than in dizygotic twins,^5^ which indicates significant, but not exclusive, genetic contributions. Epidemiological studies indicate that oral cleft phenotypes may have different underlying etiologies. For instance, isolated CP and CL/P seldom occur in the same family.^3^ Siblings of patients with CL/P have an increased frequency of CL/P but not of isolated CP, while siblings of patients with isolated CP have an increased frequency of isolated CP but not of CL/P.^3^ Moreover, CL/P and CP display different sex ratios and prevalence among oral cleft phenotypes. The recurrence risk of CP among siblings is higher in females than in males whereas the reverse is true for CL/P.^3, 6^ Gaining insight into the different causative etiologies of oral clefts is important as it may lead to improved diagnosis, counseling, and preventive health treatments.

Oral clefts in humans are associated with a large number of genetic diseases/syndromes,^7^ and findings from studies using genetically engineered mice with oral cleft have improved our understanding of palatogenesis.^8, 9^ As a result, many genetic mutations associated with human and mouse oral clefts have been identified and molecular functions have been elucidated. Since the identification and functional classification of disease-causing genes can reveal general biological mechanisms underlying human diseases and disorders,^10^ investigating the functional annotation of candidate genes associated with oral clefts would aid in a better understanding not only of the biological basis of these phenotypically variable and complex group of conditions but also of their underlying genetic causes.

## Materials and methods

### Genes associated with human oral cleft phenotypes

Online Mendelian Inheritance in Man (OMIM) (http://omim.org)^11^ is a comprehensive, well-established database of human genes and genetic disorders integrating genetic information with clinical phenotypes and diseases in humans. Similarly, the GATACA database (https://gataca.cchmc.org/gataca/) provides links between genes and different diseases or phenotypes using cross mapping to identify genetic overlap between different biological elements, functions, or processes. In our evaluation of the genetic basis of human palatogenesis, we first investigated congenital disorders or syndromes associated with oral clefts and their candidate genes using OMIM and GATACA. The databases were searched using the terms “cleft lip and palate”, “cleft lip/palate”, “cleft lip and/or palate”, “cleft lip”, “cleft of upper lip”, “cleft palate”, “cleft secondary palate”, “incomplete cleft palate”, “submucosal cleft palate”, “submucous cleft palate”, “soft palate cleft”, “cleft of the soft palate”, “soft cleft palate”, “cleft uvula”, and “bifid uvula”. The search was completed on 9 February 2016. In our identification of oral cleft phenotypes in humans, our primary search results were screened using the following exclusion criteria: (1) genes associated with oral clefts in mice with no evidence of association in humans; and (2) genes specifically associated with an absent uvula. The resulting list of genes associated with nonsyndromic and syndromic oral cleft phenotypes in humans was used for ontology analysis. Positive hits were further interrogated to identify oral cleft subphenotypes through review of either the Clinical Synopsis or articles cited in OMIM (Supplementary Table 1 and Supplementary References). OMIM and the NCBI Gene database (http://www.ncbi.nlm.nih.gov/gene) were used to identify the corresponding proteins and Entrez Gene ID of each gene.

### Gene ontology analysis

For a better understanding of the genetic contributions underlying oral clefts, genes associated with oral cleft were further analyzed based on biological process, molecular function, and gene family using the Protein ANalysis THrough Evolutionary Relationships (PANTHER) database (http://pantherdb.org).^12^ Briefly, Entrez Gene IDs were uploaded to identify unique and annotated genes for inclusion in the ontology analysis. The resulting gene lists were evaluated using tests for enrichment that identify functional classes in which the genes of a given class have values that are non-randomly selected from a genome-wide distribution of values.^12^ Statistically significant enrichment of the data set in a given process was determined using binomial testing with Bonferroni corrections for multiple testing as described previously.^13^ Only those classes demonstrating statistically significant (*P*< 0.05) enrichment were used for gene family analysis. Putative chemical–gene–disease interactions were identified using the Comparative Toxicogenomics Database (CTD) (http://ctdbase.org).^14^ For CTD analysis, derived nominal *P*-values were adjusted using the false discovery rate as described by Benjamini and Yekutieli.^15^ The CTD contains many classes with similar protein constituents. Therefore, the gene counts of those classes that were a complete subset of another were discarded.

## Results

### Gene profiles differ depending on the oral cleft phenotype

**Figure 1:**
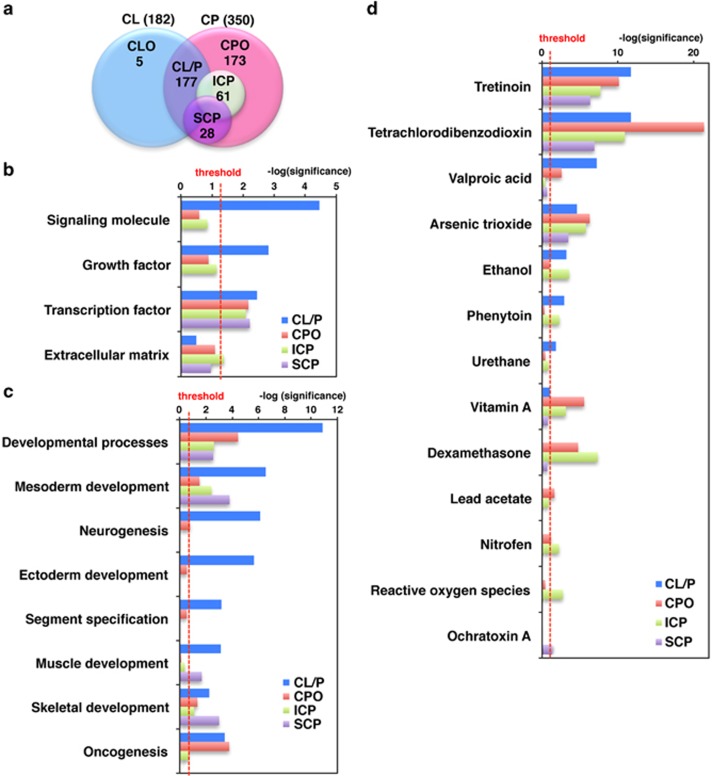
**Gene profiles differ depending on cleft palate phenotype**. (**a**) The overlap between human genes associated with cleft phenotypes is depicted in the Venn diagram. The numbers in each area represent the gene count for the particular section. (**b**–**d**) Gene ontology analysis of genes associated with human cleft palate phenotypes according to molecular function (**b**), biological process (**c**) and chemicals (**d**). Plotted is the –log(*P*-value) with the threshold set to 1.3 [log(0.05)]. CP, cleft palate; CL/P, cleft lip and/or palate; CPO, cleft palate only; ICP, incomplete cleft palate; SCP, submucous cleft palate; CL, cleft lip; CLO, cleft lip only.

As a result of our search using OMIM and GATACA (refer to Materials and Method section for a full list of search terms), we found over 350 candidate genes having one or more syndromic and/or nonsyndromic oral cleft annotations (Supplementary Table 1). Since phenotypic classification of human genes often yields important insights into gene function,^16^ we classified the identified genes based on their association with CL/P, CP only (CPO), incomplete CP, and submucous CP as shown in Figure 1a.

To investigate whether gene profiles differ among oral cleft phenotypes, we performed a gene ontology analysis first comparing candidate genes using the PANTHER database (Figure 1b and 1c and Table 1, 2, 3). Based on studies that investigated expression patterns and phenotypes in mutant mice, homeobox transcription factors have roles in the patterning of the upper and lower jaws.^17, 18^ We found that when genes were analyzed according to molecular function, those found in the transcription factor category, especially those genes that contain a homeobox transcription domain, were enriched in all oral cleft phenotypes (Figure 1b, Table 1, family #1 in Table 3). We also found that genes associated with signaling molecules (*P*=0.000035) and growth factor (*P*=0.0015) were significantly enriched in CL/P, and genes associated with the extracellular matrix were significantly enriched in incomplete CP (*P*=0.042) (Figure 1b and Table 1). When genes were analyzed according to biological process, neurogenesis (*P*=0.00000076), ectoderm development (*P*=0.0000021), and segment specification (*P*=0.00066) were enriched in only CL/P (Figure 1c and Table 2). In submucous CP, we found that muscle development (*P*=0.0021) and skeletal development (*P*=0.00099) were enriched (Figure 1c and Table 2). Developmental process and mesoderm development were significantly enriched in all oral cleft phenotypes (Figure 1c).

We next investigated possible chemical–gene–disease interactions using the CTD to investigate the mechanisms underlying environmentally influenced oral clefts. We found that the enrichment distribution of chemicals was also different among cleft phenotypes (Figure 1d). Tretinoin (the carboxylic acid form of vitamin A), tetrachlorodibenzodioxin (also known as Dioxin), and arsenic trioxide (an anti-cancer chemotherapy drug) were significantly enriched in all oral cleft phenotypes (Figure 1d). Valproic acid, a medication primarily used to treat epilepsy and bipolar disorder, was significantly enriched in CL/P (*P*=0.00000006144) and CPO (*P*=0.002719), but not in incomplete CP and submucous CP (Figure 1d). In addition, we found that ethanol and phenytoin (an anti-seizure medication) were both enriched in CL/P and incomplete CP (Figure 1d), whereas vitamin A and dexamethasone (a corticosteroid) were both enriched in CPO and incomplete CP (Figure 1d). The herbicide nitrofen and reactive oxygen species were significantly enriched in incomplete CP, whereas ochratoxin A, which is a mycotoxin produced by *Aspergillus ochraceus*, was enriched specifically in submucous CP (Figure 1d).

We also analyzed genes according to gene family. Interestingly, gene products involved in the TGF-β signaling pathway (family #4 in Table 3) were enriched in CPO (*P*=0.00024) and incomplete CP (*P*=0.00019) whereas genes involved in the fibroblast growth factor (FGF) family were only enriched in CL/P (*P*=0.0000032) (family #5 in Table 3). In addition, we found that all three of the T-box protein, collagen-α chain protein, and TGF-β families were associated with CPO and incomplete CP (families #2–4 in Table 3).

## Discussion

Palatogenesis involves many diverse genes in a complex process. Oral cleft phenotypes develop when this process is disrupted in some manner because of gene dysfunction. However, oral cleft phenotypes can vary significantly, and this phenotypic variation likely reflects the involvement of different genes and/or changes in the functional contributions of the same genes. To understand better the genetic contributions underlying different oral cleft phenotypes, it is necessary to identify and characterize these culprit genes. It is known that the empirical recurrence risks for CP and CL/P are independent, characterized by differences in sex ratios and prevalence.^3^ Similarly, our ontology analysis found different gene profiles indicating different underlying genetic etiologies of CP and CL/P. When genes were analyzed according to molecular function, biological process, chemical–gene–disease interactions, and gene family, we found distinct genetic profiles for different cleft palate phenotypes such as CL/P, CP, incomplete CP, and submucous CP. The results of our gene ontology analyses support the findings of earlier epidemiological studies that suggest that different genetic etiologies underlie different oral cleft phenotypes. They further demonstrate the usefulness of ontological candidate gene analysis in understanding gene function in palatogenesis.

Using ontology analysis, we found that the T-box protein family, the collagen-α chain protein family, and the TGF-β family were associated with CPO and incomplete CP. Consistent with our findings, a study reported that TGF-β regulates collagen synthesis and degradation, thereby affecting the amount of collagen present in the mesenchyme of the embryonic palate.^19^ The T-box gene, *TBX1,* is the major candidate gene for DiGeorge syndrome (OMIM #188400) and may be responsible for several phenotypes including cleft palate, while mutations in *TBX22* cause a form of X-linked cleft palate (OMIM #303400). Similarly, mutations in the collagen-α chain genes, *COL2A1*, *COL9A2*, *COL11A1*, and *COL11A2*, have been associated with different forms of Stickler syndrome (OMIM#108300, #614284, #604841, and #184840, respectively), a clinically variable condition that includes cleft palate. As disruption of T-box proteins and collagen-α chain proteins both contribute to CPO and incomplete CP in humans, and that *Tbx1* knockout mice exhibit different CP phenotypes including incomplete CP and submucosal CP,^20^ further investigations to determine whether deletion of *Tbx1* or *Tbx22* affects expression of collagen-α chain genes in mouse palatal shelves are warranted.

In summary, we identified a pool of candidate genes associated with different oral cleft phenotypes. Our gene ontology analysis revealed that genes associated with each cleft palate phenotype show different functional profiles. It is possible that some of the candidate genes identified are involved in tongue or bone anomalies and induce oral clefts during palatogenesis as a secondary defect. In addition, some polymorphisms identified in listed genes may not be disease-causing *per se*, but benign sequence variants in linkage disequilibrium with pathogenic variants. In addition to gene mutations, epigenetic changes and microRNA regulation may alter gene expression during palatogenesis. Nevertheless, the results of the gene ontology analysis indicated distinct genetic profiles for each oral cleft phenotype and differences in the underlying genetic etiologies of oral clefts. Analysis of the candidate genes and their products may provide an opportunity to discover new disease-causing genes implicated in palatogenesis.

## Figures and Tables

**Table 1:** Classification of candidate genes associated with human oral cleft phenotypes according to molecular function

Molecular function	Cleft type	%	*P*-value	Genes
Signaling molecule	CL/P	12.4	3.5 × 10^−5^	ANK1, BMP4, EFNB1, FGF1*, FGF10, FGF17, FGF19*, FGF2*, FGF8, GRIP1, IL1RN, IL1B, JAG2*, NOG*, PDGFC*, SEMA3E, SHH, SPRY2*, TGFA, WNT3, WNT5A, WNT7A
	CPO	6.4	2.6 × 10^−1^	*STAMBP, BMP2, CRLF1, EDN1, GNRH1, GDF1, GDF6, PLCB4, SPRY4, TGFB2, TGFB3*
	ICP	9.8	1.4 × 10^−1^	*BMP4, CRLF1, EDN1, GDF1, TGFB2, TGFB3*
	SCP	0.0	N/A	N/A
Growth factor	CL/P	4.0	1.5 × 10^−3^	FGF1*, FGF10, FGF17, FGF19*, FGF2*, FGF8, PDGFC*
	CPO	2.3	1.3 × 10^−1^	*GDF1, GDF6, TGFB2, TGFB3*
	ICP	4.9	7.1 × 10^−2^	*GDF1, TGFB2, TGFB3*
	SCP	0.0	N/A	N/A
Transcription factor	CL/P	18.6	3.6 × 10^−3^	ARNT*, ALX1, ALX3, GATA3, GLI2, GLI3, LHX8*, LMX1B, SMAD4, TBX10*, TGIF1, YAP1, ZIC2, ARX*, DLX5, ESR1*, FOXE1, GRHL3, IRF6, JAG2*, MED12, MEOX1, MSX1, MSX2, PAX3, PAX7*, RARA*, SPRY2*, TFAP2A, TP63, MAFB*, SKI, VAX1
	CPO	18.5	6.8 × 10^−3^	ALX4, CTCF, FEZF1, GATA6, KAT6B, MKX*, NKX2-5, NKX2-6, SATB2, SMAD3, SOX2, SOX9, TBX1, TBX15, TBX22, TBX4, WT1, ZIC3, FOXC2, HOXA2, OTX2, PRRX1, PITX1, PQBP1, RB1, RAI1, RARB, RUNX2, SRY, SPRY4, TWIST1, ZEB2
	ICP	9.8	8.3 × 10^−3^	*GATA6, GLI3, NKX2-5, NKX2-6, SATB2, SMAD3, TBX1, TBX22, DLX5, IRF6, TP63*
	SCP	32.1	6.1 × 10^−3^	*GATA6, NKX2-5, NKX2-6, TBX1, TBX22, DLX5, MED12, RUNX2, ZEB2*
Transferase	CL/P	6.2	3.6 × 10^−1^	MTR, NAT1*, NAT2*, NEK1, WHSC1, B3GLCT, ESCO2, GSTT1*, LARGE, POMT1, POMT2
	CPO	8.7	5.0 × 10^−2^	GMPPB, KAT6B, B3GALT6, ALG3, B3GAT3, CHST14, COMT*, CHSY1, PSAT1, HS6ST1, KMT2D, NSD1, PTDSS1, POLR1D, XYLT1
	ICP	3.3	9.6 × 10^−1^	ALG3, COMT*
	SCP	0.0	N/A	N/A
Extracellular matrix	CL/P	3.4	3.2 × 10^−1^	MKS1, COL8A1*, FLRT3, MMP9*, NTN1*, NOG*
	CPO	4.6	8.1 × 10^−2^	*BMPER, COL2A1, COL9A2, COL11A1, COL11A2, GPC3, MEGF10, TNXB*
	ICP	8.2	4.2 × 10^−2^	*COL2A1, COL11A1, COL11A2, GPC3, TNXB*
	SCP	10.7	1.1 × 10^−1^	*COL11A1, COL11A2, GPC3*

CL/P, cleft lip and/or palate; CPO, cleft palate only; ICP, incomplete cleft palate; SCP, submucous cleft palate; N/A, not applicable; %, involved genes/total genes × 100; *P*-value, probabilities were adjusted for multiple comparisons across all PANTHER molecular functions using Bonferroni correction.

* Genes associated with nonsyndromic oral clefts.

**Table 2:** Classification of candidate genes associated with human oral cleft phenotypes according to biological process

Biological process	Cleft type	%	*P*-value	Genes
Developmental processes	CL/P	31.6	1.3 × 10^−11^	ALX1, ALX3, CDON, GATA3, GLI2, GLI3, LMX1B, RYK*, SMAD4, TBX10*, WDR35, ZIC2, ALPL, ARX*, BMP4, DLX5, EFNB1, ESR1*, EYA1, FGF1*, FGF19*, FGF2*, FGFR1, FGFR2, FGFR3, FOXE1, FKTN, JAG2*, LHX8*, MEOX1, MID1, MSX1, MSX2, MYH3, MYH9*, NTN1*, NOG*, PAX3, PAX7*, PDGFC*, PAFAH1B1, PORCN, RARA*, SEMA3E, SHH, SPRY2*, SUFU, TFAP2A, TPM2, TNNI2, MAFB*, SKI, VAX1, WNT3, WNT5A, WNT7A
	CPO	24.3	3.6 × 10^−5^	ALX4, BMPER, GATA6, L1CAM, KAT6B, NKX2-5, NKX2-6, SMAD3, TBX1, TBX15, TBX22, TBX4, TCOF1, WT1, ZIC3, BMP2, BUB1B, COL9A2, COL11A1, COL11A2, FLVCR2, FOXC2, GNRH1, GNRHR, GDF1, HOXA2, LRP4, MEGF10, OTX2, PRRX1, PDGFRA*, PTPN11, RB1, RARB, RUNX2, SPRY4, SMC1A, TNXB, TGFB2, TGFB3, TGFBR2, TWIST1
	ICP	27.9	2.5 × 10^−3^	*GATA6, GLI3, NKX2-5, NKX2-6, SMAD3, TBX1, TBX22, BMP4, COL11A1, COL11A2, EYA1, FGFR2, GDF1, TNXB, TGFB2, TGFB3, TGFBR2*
	SCP	35.7	2.8 × 10^−3^	*GATA6, NKX2-5, NKX2-6, TBX1, TBX22, COL11A1, COL11A2, DLX5, FGFR1, RUNX2*
Mesoderm development	CL/P	12.4	2.9 × 10^−7^	ALX1, CDON, GATA3, GLI3, ALPL, BMP4, DLX5, FGF1*, FGF2*, FOXE1, FKTN, MEOX1, MSX1, MSX2, MYH3, MYH9*, NOG*, SPRY2*, SUFU, TPM2, TNNI2, SKI
	CPO	6.9	3.1 × 10^−2^	*NKX2-5, NKX2-6, TBX22, TBX4, BMP2, COL9A2, COL11A1, COL11A2, FOXC2, GDF1, RUNX2, SPRY4*
	ICP	13.1	3.8 × 10^−3^	*GLI3, NKX2-5, NKX2-6, TBX22, BMP4, COL11A1, COL11A2, GDF1*
	SCP	25.0	1.6 × 10^−4^	*NKX2-5, NKX2-6, TBX22, COL11A1, COL11A2, DLX5, RUNX2*
Neurogenesis	CL/P	12.4	7.6 × 10^−7^	ALX3, GLI3, RYK*, WDR35, ZIC2, ARX*, EFNB1, FGF19*, FGFR1, FGFR2, FGFR3, FOXE1, JAG2*, LHX8*, NTN1*,PAX3, PAX7*, PAFAH1B1, SEMA3E, MAFB*, SKI, VAX1
	CPO	5.8	1.6 × 10^−1^	*ALX4, L1CAM, TCOF1, ZIC3, FOXC2, HOXA2, MEGF10, OTX2, PRRX1, TNXB*
	ICP	4.9	6.4 × 10^−1^	*GLI3, FGFR2, TNXB*
	SCP	3.6	1.0	*FGFR1*
Ectoderm development	CL/P	13.0	2.1 × 10^−6^	ALX3, GLI3, RYK*, WDR35, ZIC2, ARX*, EFNB1, FGF19*, FGFR1, FGFR2, FGFR3, FOXE1, JAG2*, LHX8*, NTN1*, PAX3, PAX7*, PAFAH1B1, SEMA3E, TFAP2A, MAFB*, SKI, VAX1
	CPO	5.8	2.8 × 10^−1^	*ALX4, L1CAM, TCOF1, ZIC3, FOXC2, HOXA2, MEGF10, OTX2, PRRX1, TNXB*
	ICP	4.9	7.2 × 10^−1^	*GLI3, FGFR2, TNXB*
	SCP	3.6	1.0	*FGFR1*
Segment specification	CL/P	4.0	6.6 × 10^−4^	ALX3, ARX*, DLX5, FOXE1, PAX3, PAX7*, PORCN
	CPO	1.7	2.9 × 10^−1^	*ALX4, FOXC2, HOXA2*
	ICP	0.0	N/A	N/A
	SCP	3.6	1.0	*DLX5*
Skeletal development	CL/P	4.5	3.8 × 10^−4^	ALX1, ALPL, BMP4, DLX5, MSX1, MSX2, NOG*, SUFU
	CPO	2.9	4.5 × 10^−2^	*NKX2-5, NKX2-6, BMP2, COL9A2, RUNX2*
	ICP	4.9	7.9 × 10^−2^	*NKX2-5, NKX2-6, BMP4*
	SCP	14.3	9.9 × 10^−4^	*NKX2-5, NKX2-6, DLX5, RUNX2*
Muscle development	CL/P	4.5	7.3 × 10^−4^	CDON, DLX5, FKTN, MYH3, MYH9*, TPM2, TNNI2, SKI
	CPO	1.2	7.8 × 10^−1^	*NKX2-5, NKX2-6*
	ICP	3.3	4.1 × 10^−1^	*NKX2-5, NKX2-6*
	SCP	10.7	2.1 × 10^−2^	*NKX2-5, NKX2-6, DLX5*
Oncogenesis	CL/P	6.8	5.6 × 10^−3^	SMAD4, FGFR1, FGFR2, FGFR3, IRF6, RARA*, SHH, SUFU, TFAP2A, TP63, KRAS, SKI
	CPO	8.7	1.7 × 10^−4^	BRIP1, SMAD3, WT1, BUB1B, CDKN1C, LOXL3, NRAS, PTEN, PDGFRA*, RB1, RARB, RUNX2, ST5*, HRAS, BRAF
	ICP	6.6	2.1 × 10^−1^	*SMAD3, FGFR2, IRF6, TP63*
	SCP	10.7	1.5 × 10^−1^	*FGFR1, RUNX2, BRAF*

CL/P, cleft lip and/or palate; CPO, cleft palate only; ICP, incomplete cleft palate; SCP, submucous cleft palate; N/A, not applicable; %, involved genes/total genes × 100; *P*-value, probabilities were adjusted for multiple comparisons across all PANTHER molecular functions using Bonferroni correction.

* Genes associated with nonsyndromic oral clefts.

**Table 3:** Classification of candidate genes associated with human oral cleft phenotypes according to gene family

	*#*	*Domain name*	*Cleft type*	*%*	P*-value*	*Genes*
**CL/P, CPO and SCP**	**1**	Homeobox protein	CL/P	8.7	3.1 × 10^−5^	ALX1, ALX3, ARX*, DLX5, MSX1, MSX2, PAX3, PAX7*, VAX1, LHX8*, MEOX1, TGIF1, LMX1B, SIX3,
			CPO	5.5	4.9 × 10^−3^	ALX4, HOXA2, MKX*, *NKX2-5, NKX2-6, OTX2, PITX1, PRRX1, SATB2, SIX3, ZEB2*
			SCP	13.4	2.6 × 10^−2^	*DLX5, NKX2-5, NKX2-6, SIX3, ZEB2*
**CPO, ICP and SCP**	**2**	T-box protein	CPO	2.3	4.9 × 10^−4^	*TBX1, TBX15, TBX22, TBX4*
			ICP	3.3	5.6 × 10^−2^	*TBX1, TBX22*
			SCP	7.1	2.5 × 10^−2^	*TBX1, TBX22*
**CPO and ICP**	**3**	Collagen alpha chain	CPO	2.3	5.0 × 10^−2^	*COL2A1, COL9A2, COL11A1, COL11A2*
			ICP	4.9	3.7 × 10^−2^	*COL2A1, COL11A1, COL11A2*
	**4**	TGF-β family	CPO	2.9	2.4 × 10^−4^	*GDF6, GDF1, TGFB2, TGFB3, BMP2*
			ICP	6.6	1.9 × 10^−4^	*GDF1, TGFB2, TGFB3, BMP4*
**CL/P only**	**5**	Heparin-binding FGF family member	CL/P	3.4	3.2 × 10^−6^	FGF17, FGF1*, FGF2*, FGF8, FGF10, FGF19*
	**6**	Patched-related	CL/P	1.7	4.6 × 10^−3^	PTCH1, PTCH2, DISP1*
	**7**	Zinc finger protein Zic and Gli	CL/P	1.7	8.5 × 10^−3^	*GLI2, GLI3, ZIC2*
	**8**	Neurotransmitter gated ion channel	CL/P	2.2	8.7 × 10^−3^	GABRB3*, CHRNA1, CHRND, CHRNG
	**9**	Tyrosine protein kinase	CL/P	2.8	1.1 × 10^−2^	FGFR1, FGFR2, FGFR3, RYK*, ROR2
	**10**	Wnt related	CL/P	1.7	1.4 × 10^−2^	*WNT3, WNT5A, WNT7A*
	**11**	N-hydroxyarylamine o-acetyltransferase	CL/P	1.1	1.9 × 10^−2^	NAT1*, NAT2*
	**12**	IFT140/172-related	CL/P	1.1	1.9 × 10^−2^	*IFT140, IFT172*
	**13**	Dolichyl-phosphate-mannose-protein mannosyltransferase	CL/P	1.1	3.7 × 10^−2^	*POMT1, POMT2*
	**14**	MTR related	CL/P	1.1	4.6 × 10^−2^	*MTHFR, MTR*
	**15**	Tropomyosin	CL/P	1.1	4.6 × 10^−2^	MYH9*, TMP2
**CPO only**	**16**	Sox transcription factors	CPO	1.7	1.6 × 10^−2^	*SOX2, SOX9, SRY*
	**17**	Origin of replication binding protein	CPO	1.2	1.9 × 10^−2^	*CDC6, ORC1*
**ICP only**	**18**	TGF-β receptor type I and II	ICP	3.3	4.0 × 10^−2^	*TGFBR1, TGFBR2*

CL/P, cleft lip and/or palate; CPO, cleft palate only; ICP, incomplete cleft palate; SCP, submucous cleft palate; TGF, transforming growth factor; FGF, fibroblast growth factor; MTR, Methyltetrahydrofolate-homocysteine methyltransferase; %: involved genes/total genes × 100; *P*-value, probabilities were adjusted for multiple comparisons across all PANTHER molecular functions using Bonferroni correction.

* Genes associated with nonsyndromic oral clefts.
