# Supplementary material for: Identification of shared and unique gene families associated with oral clefts
Source: Int J Oral Sci. 2017 Jan 20;9(2):104–9. doi: 10.1038/ijos.2016.56 (PMC5518969; doi:10.1038/ijos.2016.56)
Supplement: Supplementary Table 1 [file ijos201656x1.pdf]

Supplementary Information

Supplementary Table 1. Candidate genes for human oral clefts

Humans							Mutant mice
Gene	Protein	Cytogenetic Location	Syndrome	OMIM Number	Entrez Gene ID	Cleft Phenotypes	Cleft Phenotypes
ABCA4	ATP-binding cassette, subfamily A, member 4	1p22.1	-	*601691	24	CL/P [1]	n.r.
ABCB1	ATP-binding cassette, sub-family B, member 1	7q21.12	-	*171050	5243	CL/P [2]	n.r.
ACTB	actin, beta	7p22.1	Baraitser-Winter syndrome 1<br>Dystonia, juvenile-onset	*102630	60	CL/P, BU	n.r.
ADAMTS20	disintegrin-like and metalloproteinase with thrombospondin type 1 motif, 20	12q12	-	*611681	80070	CL/P [3]	CP [4]
AFA	ankyloblepharon filiforme adnatum	n.r.	Ankyloblepharon filiforme adnatum cleft palate	106250	170	CL/P	n.r.
ALG3	ALG3, alpha-1,3-mannosyltransferase	3q27.1	Congenital disorder of glycosylation, type Id	*608750	10195	BU	n.r.
ALPL	alkaline phosphatase, liver/bone/kidney	1p36.12	Hypophosphatasia, infantile	*171760	249	CL/P	n.r.
ALX1	aistaless-like homeobox 1	12q21.31	Frontonasal dysplasia 3	*601527	8092	CL/P	CP [5]
ALX3	aristaless-like homeobox 3	1p13.3	Frontonasal dysplasia 1	*606014	257	CL/P	CP [6]
ALX4	aristaless-like homeobox 4	11p11.2	Frontonasal dysplasia 2<br>Parietal foramina 2<br>Craniosynostosis 5	*605420	60529	Cleft alae nasi, CP	CP [6]
AMER1	APC membrane recruitment protein 1	Xq11.2	Osteopathia striata with cranial sclerosis	*300647	139285	CL/P, BU	n.r.
ANK1	ankyrin 1	8p11.21	Spherocytosis, type 1	*612641	286	CL/P	n.r.
ANOS1 /KAL1	anosmin 1	Xp22.31	Hypogonadotropic hypogonadism 1 with or without anosmia	*300836	3730	CL/P	n.r.
AOCH	acromegaloid features, overgrowth, cleft palate, and hernia	Chr.11	Acromegaloid features, overgrowth, cleft palate, and hernia	%606049	93956	CP	n.r.
ARHGAP31	Rho GTPase activating protein 31	3q13.33	Adams-Oliver syndrome 1	*610911	57514	CL/P	n.r.
ARID1B	AT-rich interaction domain-containing protein 1B	6q25.3	Coffin-Siris syndrome 1	*614556	57492	CP	n.r.
ARNT	aryl hydrocarbon receptor nuclear translocator	1q21.3	-	*126110	405	CL/P [7]	n.r.
ARX	aristaless-related homeobox, X-linked	Xp21.3	-	*300382	170302	CL/P (rare) [8]	n.r.
ASXL1	additional sex combs-like 1	20q11.21	Bohring-opitz syndrome	*612990	171023	CL/P	CP [9]
ATR	ATR serine/threonine kinase	3q23	Seckel syndrome 1	*601215	545	CP	n.r.
ATRX	alpha thalassemia/mental retardation syndrome X-linked	Xq21.1	Mental retardation-hypotonic facies syndrome, X-linked, 1	*300032	546	CP	n.r.
AUTS2 /KIAA0442	autism susceptibility candidate 2	7q11.22	Mental retardation, autosomal dominant 26	*607270	26053	CL	n.r.
B3GALT6	beta-1,3-galactosyltransferase 6	1p36.33	Ehlers-Danlos syndrome, progeroid type, 2<br>Spondyloepimetaphyseal dysplasia with joint laxity, type 1	*615291	126792	CP (rare)	n.r.
B3GAT3	beta-1,3-glucuronyltransferase 3	11q12.3	Multiple joint dislocations, short stature, craniofacial dysmorphism, and congenital heart defects	*606374	26229	CP (rare)	n.r.
B3GLCT /B3GALTL	beta 3-glucosyltransferase	1p36.33	Peters-plus syndrome	*610308	145173	CL/P	n.r.
BCL3	B-cell leukemia/lymphoma 3	19q13.32	-	*109560	602	CL/P [10]	n.r.
BCOR	BCL6 corepressor	Xp11.4	Oculofaciocardiodental<br>Microphthalmia, syndromic 2	*300485	54880	CP, BU, SCP	n.r.
BMP2	bone morphogenetic protein 2	20p12.3	Brachydactyly, type A2	*112261	650	CP	CP [11]
BMP4	bone morphogenetic protein 4	14q22.2	Microphthalmia, syndromic 6<br>Orofacial Cleft 11	*112262	652	CL/P, BU	CP [12,13]
BMPER	BMP binding endothelial regulator	7p14.3	Diaphanospondylodysostosis	*608699	168667	CP	n.r.
BRAF	B-Raf proto-oncogene, serine/threonine kinase	7q34	Cardiofaciocutaneous syndrome 1	*164757	673	CP, SCP	n.r.
BRIP1	BRCA1-interacting protein 1	17q23.2	Fanconi anemia, complementation group J	*605882	83990	CP	n.r.
BUB1B	BUB1 mitotic checkpoint serine/threonine kinase B	15q15.1	Mosaic variegated aneuploidy syndrome 1	*602860	701	CP	n.r.
C2CD3	C2 calcium-dependent domain containing 3	11q13.4	Orofaciodigital syndrome XIV	*615944	26005	CP	n.r.

C5ORF42	chromosome 5 open reading frame 42	5p13.2	Orofaciodigital syndrome VI	*614571	65250	CL/P	n.r.
CASK	calcium/calmodulin-dependent serine protein kinase	Xp11.4	Mental retardation and microcephaly with pontine and cerebellar hypoplasia	*300172	8573	CP	CP [14,15]
CC2D2A	coiled-coil and C2 domain containing 2A	4p15.32	COACH syndrome<br>Joubert syndrome 9<br>Meckel syndrome 6	*612013	57545	CL/P	CP [MGI]
CD96	CD96 molecule	3q13.1-q13.2	C syndrome	*606037	10225	CL/P	n.r.
CDC6	cell division cycle 6	17q21.2	Meier-Gorlin syndrome 5	*602627	990	CP, SCP	n.r.
CDH1	cadherin 1	16q22.1	Gastric cancer, hereditary diffuse	*192090	999	CL/P	n.r.
CDKN1C	cyclin-dependent kinase inhibitor 1C	11p15.4	Intrauterine growth retardation, metaphyseal dysplasia, adrenal hypoplasia congenita, and genital anomalies<br>Beckwith-Wiedemann syndrome	*600856	1028	CP	CP [16,17]
CDON	cell adhesion molecule-related/downregulated by oncogenes	11q24.2	Holoprosencephaly 11	*608707	50937	CL/P	CP [18]
CHD7	chromodomain helicase DNA-binding protein 7	8q12.1-q12.2	CHARGE syndrome<br>Hypogonadotropic hypogonadism 5 with or without anosmia	*608892	55636	CL/P	CP[19,20]
CHRNA1	cholinergic receptor nicotinic alpha 1 subunit	2q31.1	Multiple pterygium syndrome, lethal type	*100690	1134	CL/P	n.r.
CHRND	cholinergic receptor nicotinic delta subunit	2q37.1	Multiple pterygium syndrome, lethal type	*100720	1144	CL/P	n.r.
CHRNG	cholinergic receptor nicotinic gamma subunit	2q37.1	Multiple pterygium syndrome, lethal type<br>Multiple pterygium syndrome, Escobar variant	*100730	1146	CL/P	n.r.
CHST14	carbohydrate sulfotransferase 14	15q15.1	hlers-Danlos syndrome, musculocontractural type 1	*608429	113189	CP	n.r.
CHSY1	chondroitin sulfate synthase 1	15q26.3	Temtamy preaxial brachydactyly syndrome	*608183	22856	CP (rare)	n.r.
CKAP2L	cytoskeleton associated protein 2 like	2q13	Filippi syndrome	*616174	150468	CP	n.r.
CLPTM1	cleft lip and palate-associated transmembrane protein 1	19q13.32	-	*604783	1209	CL/P, SCP? [21]	n.r.
COL11A1	collagen, type XI, alpha-1	1p21.1	Fibrochondrogenesis 1<br>Marshall syndrome<br>Stickler syndrome, type II	*120280	1301	CP, BU, SCP	CP [22]
COL11A2	collagen, type XI, alpha-2	6p21.32	Stickler syndrome, type III<br>Weissenbacher-Zweymuller syndrome<br>Otospondylomegaepiphyseal dysplasia	*120290	1302	CP, BU IPC, SCP	n.r.
COL2A1	collagen, type II, alpha-1	12q13.11	Otospondylomegaepiphyseal dysplasia<br>Achondrogenesis, type II<br>Kniest dysplasia<br>Spondyloepimetaphyseal dysplasia, Strudwick type<br>Spondyloepiphyseal dysplasia congenital<br>Stickler syndrome, type I<br>Stickler syndrome, type I, nonsyndromic ocular	+120140	1280	CP, BU	CP [23]
COL8A1 /FILIP1L	collagen, type VIII, alpha-1	3p12.3	-	*120251	1295	CL/P [24]	n.r.
COL9A2	collagen type IX alpha 2	1p34.2	Stickler syndrome, type V	*120260	1298	CP	n.r.
COLEC11	collectin subfamily member 1	2p25.3	3MC syndrome 2	*612502	78989	CL/P	n.r.
COMT	catechol-o-methyltransferase	22q11.21	-	+116790	1312	CP, BU [25]	n.r.
CRISPLD2	cysteine-rich secretory protein, LCCL domain-containing, 2	16q24.1	-	*612434	83716	CL/P [26]	n.r.
CRLF1	cytokine receptor-like factor 1	19p13.11	Cold-induced sweating syndrome 1	*604237	9244	CP, ICP	n.r.
CTCF	CCCTC-binding factor	16q22.1	Mental retardation, autosomal dominant 21	*604167	10664	CP	n.r.
CYP26C1	cytochrome P450 family 26 subfamily C member 1	10q23.33	Focal facial dermal dysplasia 4	*608428	340665	CL/P	n.r.
DDX59	DEAD (Asp-Glu-Ala-Asp) box polypeptide 59	1q32.1	Orofaciodigital syndrome V	*615464	83479	CP, BU	n.r.
DHCR24	24-dehydrocholesterol reductase	1p32.3	Desmosterolosis	*606418	1718	CP, SCP	n.r.
DHCR7	7-dehydrocholesterol reductase	11q13.4	Smith-Lemli-Opitz syndrome	*602858	1717	CP, ICP	CP [27,28]
DHODH	dihydroorotate dehydrogenase	16q22.2	Miller syndrome	*126064	1723	CL/P	n.r.

DIH1	diaphragmatic hernia 1	15q26.1	Diaphragmatic hernia, congenital	%142340	1732	CL/P	n.r.
DIS3L2	DIS3 like 3'-5' exoribonuclease 2	2q37.1	Perlman syndrome	*614184	129563	CP (rare)	n.r.
DISP1	dispatched, drosophila, homolog of, 1	1q41	-	*607502	84976	CL/P [29]	n.r.
DLX5	distal-less homeobox 5	7q21.2-q21.3	Split-hand/foot malformation 1	*600028	1749	CL/P, CP, SCP	CP [30,31]
DOK7	docking protein 7	4p16.3	Fetal akinesia deformation sequence	*610285	285489	CP	n.r.
DUSP6	dual specificity phosphatase 6	12q21.33	Hypogonadotropic hypogonadism 19 with or without anosmia	*602748	1848	CP (rare)	n.r.
DYNC2H1	dynein, cytoplasmic 2, heavy chain 1	11q22.3	Short-rib thoracic dysplasia 3 with or without polydactyly	*603297	79659	CL/P	n.r.
EARS2	glutamyl-tRNA synthetase 2, mitochondrial	16p12.2	Combined oxidative phosphorylation deficiency 12	*612799	124454	CP, ICP	n.r.
ECEL1	endothelin-converting enzyme-like 1	2q37.1	Arthrogryposis, distal, type 5d	*605896	9427	CP, SCP	n.r.
EDN1	endothelin 1	6p24.1	Auriculocondylar syndrome 3	+131240	1906	BU	CP [32]
EEC1	ectrodactyly, ectodermal dysplasia and cleft lip/palate syndrome 1	7q11.2-q21.3	Ectrodactyly, Ectodermal Dysplasia, And Cleft Lip/Palate Syndrome 1	%129900	1913	CL/P	n.r.
EFNB1	ephrin b1	Xq13.1	Craniofrontonasal dysplasia	*300035	1947	CL/P	CP [33,34]
EFTUD2	elongation factor Tu GTP-binding domain-containing 2	17q21.31	Mandibulofacial dysostosis, guion-almeida type	*603892	9343	CP, BU	n.r.
EIF4A3 /DDX48	eukaryotic translation initiation factor 4A3	17q25.3	Robin sequence with cleft mandible and limb anomalies	*608546	9775	CP, BU	n.r.
EPG5	ectopic P-granules autophagy protein 5 homolog	18q12.3-q21.1	Vici syndrome	*615068	57724	CL/P	n.r.
ERCC5	excision-repair, complementing defective, in Chinese hamster, 5	13q33.1	Xeroderma pigmentosum, complementation group G	*133530	2073	CP	n.r.
ESCO2	establishment of cohesion 1, s. Cerevisiae, homolog of, 2	8p21.1	Roberts syndrome<br>Sc phocomelia syndrome	*609353	157570	CL/P	n.r.
ESR1 (?)	estrogen receptor 1	6q25.1-q25.2	-	+133430	2099	CL/P [35]	n.r.
EVC	Ellis van Creveld protein	4p16.2	Ellis-van Creveld syndrome<br>Weyers acrodental dysostosis	*604831	2121	CL	n.r.
EVC2	Ellis van Creveld syndrome 2		Ellis-van Creveld syndrome<br>Weyers acrodental dysostosis	*607261	132884	CL	n.r.
EYA1	eyes absent 1	8q13.3	Branchiootorenal syndrome 1	*601653	2138	CL/P, BU	CP [36]
FAF1	Fas-associated factor 1	1p32.3	Orofacial cleft 13	*604460	11124	CP	n.r.
FAM20C	family with sequence similarity 20	7p22.3	Raine syndrome	*611061	56975	CP	n.r.
FEZF1	FEZ family zinc finger 1	7q31.32	Hypogonadotropic hypogonadism 22 with or without anosmia	*613301	389549	CP	n.r.
FGD1	FYVE, RhoGEF and PH domain containing 1	Xp11.22	Aarskog-Scott syndrome	*300546	2245	CL/P	n.r.
FGF1	fibroblast growth factor 1	5q31.3	-	*131220	2246	CL/P [37]	n.r.
FGF2	fibroblast growth factor 2	4q28.1	-	*134920	2247	CL/P [38]	n.r.
FGF19	fibroblast growth factor 19	11q13.3	-	*603891	9965	CL/P [38]	n.r.
FGF10	fibroblast growth factor 10	5p12	Aplasia of lacrimal and salivary glands<br>LADD syndrome	*602115	2255	CL/P [39]	CP [40,41]
FGF17	fibroblast growth factor 17	8p21.3	Hypogonadotropic hypogonadism 20 with or without anosmia	*603725	8822	CL/P	n.r.
FGF8	fibroblast growth factor 8	10q24.32	Hypogonadotropic hypogonadism 6 with or without anosmia	*600483	2253	CL/P	CL/P [42]
FGFR1	fibroblast growth factor receptor 1	8p11.23-p11.22	Pfeiffer syndrome<br>Hartsfield syndrome<br>Hypogonadotropic hypogonadism 2 with or without anosmia	*136350	2260	CL/P, SCP	CP [43]
FGFR2	fibroblast growth factor receptor 2	10q26.13	Pfeiffer syndrome<br>Apert syndrome<br>Crouzon syndrome<br>Saethre-Chotzen syndrome	*176943	2263	CL/P, BU	CP [40,44,45]
FGFR3	fibroblast growth factor receptor 3	4p16.3	Crouzon syndrome with acanthosis nigricans	*134934	2261	CL/P	n.r.
FKBP14	FK506 binding protein 14	7p14.3	Ehlers-Danlos syndrome with progressive kyphoscoliosis, myopathy, and hearing loss	*614505	55033	IPC (less common)	n.r.
FLNA	filamin A	Xq28	Frontometaphyseal dysplasia<br>Melnick-Needles syndrome<br>Otopalatodigital syndrome	*300017	2316	CP, BU	CP [46]
FLNB	filamin B	3p14.3	Atelosteogenesis<br>Larsen syndrome	*603381	2317	CL/P	n.r.

			Spondylocarpotarsal synostosis syndrome				
FLRT3	fibronectin-like domain-containing leucine-rich transmembrane protein 3	20p12.1	Hypogonadotropic hypogonadism 21 with or without anosmia	*604808	23767	CL/P	n.r.
FKRP	fukutin related protein	19q13.32	Walker-Warburg Syndrome [47]	*606596	79147	CL/P (rare)	n.r.
FKTN	fukutin	9q31.2	Walker-Warburg Syndrome [48]	*607440	2218	CL/P (rare)	n.r.
FLVCR2	feline leukemia virus subgroup C cellular receptor family member 2	14q24.3	Proliferative vasculopathy and hydranencephaly-hydrocephaly syndrome	*610865	55640	CP	n.r.
FOXC2	forkhead box C2	16q24.1	Lymphedema-distichiasis syndrome	*602402	2303	CL (4%), CP	CP [49]
FOXE1	forkhead box E1	9q22.33	Hypothyroidism, athyroidal, with spiky hair and cleft palate Bamforth syndrome	*602617	2304	CL/P [50]	CP [51]
FRAS1	fraser extracellular matrix complex subunit 1	4q21.21	Fraser syndrome	*607830	80144	CL/P	CP [52]
FREM2	FRAS1 related extracellular matrix protein 2	13q13.3	Fraser syndrome	*608945	341640	CL/P	n.r.
FTO	fat mass and obesity associated	16q12.2	Growth retardation, developmental delay, coarse facies, and early death	*610966	79068	CP, BU	n.r.
FZD4	frizzled, drosophila, homolog of, 4	11q14.2	Exudative vitreoretinopathy 1 Retinopathy of prematurity	[53]	8322	CP	n.r.
G6PC3	glucose 6 phosphatase, catalytic, 3	17q21.31	Neutropenia, severe congenital, 4, autosomal recessive	*611045	92579	CP (rare)	n.r.
GABRB3	gamma-aminobutyric acid receptor, beta-3	15q12	-	*137192	2562	CL/P [54-56]	CP [57]
GAS1	growth arrest-specific 1	9q21.33	-	*139185	2619	CL/P [58]	n.r.
GATA3	GATA binding protein 3	10p14	Hypoparathyroidism, sensorineural deafness, and renal disease	*131320	2625	CL/P	n.r.
GATA6 (?)	GATA binding protein 6	18q11.2	Persistent truncus arteriosus	*601656	2627	CP, SCP, BU	n.r.
GDF1 (?)	growth differentiation factor 1	19p13.11	Double-outlet right ventricle	*602880	2657	CP, BU	CL [59]
GDF6	growth differentiation factor 6	8q22.1	Klippel-feil syndrome 1, autosomal dominant	*601147	392255	CP	n.r.
GJA1	gap junction protein, alpha-1	6q22.31	Oculodentodigital dysplasia	*121014	2697	CL/P	n.r.
GJB2	gap junction protein beta 2	13q12.11	Vohwinkel syndrome Deafness, congenital, with keratopachydermia and constrictions of fingers and toes	*121011	2706	CP (rare)	n.r.
GLI2	GLI-Kruppel family member 2	2q14.2	Culler-Jones syndrome Holoprosencephaly 9	*165230	2736	CL/P	CP [60]
GLI3	GLI-Kruppel family member 3	7p14.1	Hypothalamic hamartomas Pallister-Hall syndrome	*165240	2737	CL/P, ICP, BU	CP [61]
GMPPB	GDP-mannose pyrophosphorylase B	3p21.31	Muscular dystrophy-dystroglycanopathy (congenital with brain and eye anomalies), type a, 14	*615320	29925	CP	n.r.
GNAI3	guanine nucleotide binding protein (G protein), alpha inhibiting activity polypeptide 3	1p13.3	Auriculocondylar syndrome 1	*139370	2773	CP, BU, SCP	n.r.
GNRH1 (?)	gonadotropin releasing hormone 1	8p21.2	?Hypogonadotropic hypogonadism 12 with or without anosmia	*152760	2796	CP	n.r.
GNRHR (?)	gonadotropin releasing hormone receptor	4q13.2	Hypogonadotropic hypogonadism 7 with or without anosmia	*138850	2798	CP	n.r.
GPC3	glypican 3	Xq26.2	Simpson-Golabi-Behmel syndrome, type 1	*300037	2719	CP, BU, SCP	n.r.
GRHL3	grainyhead-like 3	1p36.11	Van der Woude syndrome 2	*608317	57822	CL/P	CP [62] (rare)
GRIP1	glutamate receptor interacting protein 1	12q14.3	Fraser syndrome	*604597	23426	CL/P	n.r.
GSTT1	glutathione s-transferase, theta-1	22q11.23	-	*600436	2952	Orofacial cleft [63]	n.r.
GUSB	beta-glucuronidase	7q11.21	-	*611499	2990	CP [64]	n.r.
H19 (?)	H19, imprinted maternally expressed transcript (non-protein coding)	11p15.5	Beckwith-Wiedemann syndrome	*103280	283120	CP	n.r.
HDAC8	histone deacetylase 8	Xq13.1	Cornelia de Lange syndrome 5	*300269	55869	CP	n.r.
HOXA2 (#)	homeobox A2	7p15.2	Microtia, hearing impairment, and cleft palate	*604685	3199	CP	CP [65,66]
HPGD	hydroxyprostaglandin dehydrogenase 15-(NAD)	4q34.1	Hypertrophic osteoarthropathy, primary, autosomal recessive, 1	*601688	3248	CP (rare)	n.r.

HRAS	Harvey rat sarcoma viral oncogene homolog	11p15.5	Schimmelpenning-Feuerstein-Mims syndrome	*190020	3265	CP (rare)	n.r.
HS6ST1	heparan sulfate 6-o-sulfotransferase 1	2q14.3	Hypogonadotropic hypogonadism 15 with or without anosmia	*604846	9394	CP	n.r.
HYAL1	hyaluronoglucosaminidase 1	3p21.31	Mucopolysaccharidosis, type IX	*607071	3373	CP, SCP, BU	n.r.
HYLS1	hydrolethalus syndrome 1	11q24.2	Hydrolethalus syndrome 1	*610693	219844	CL/P	n.r.
ICK	intestinal cell kinase	6p12.1	Endocrine-cerebroosteodysplasia	*612325	22858	CL/P	CP [67]
ICR1 (?)	ichthyosis congenita I, erythromatous lamellar ichthyosis, ichthyosiform erythroderma	11p15.5	Beckwith-Wiedemann syndrome	*616186	3388	CP	n.r.
IFT140	intraflagellar transport 140	16p13.3	Short-rib thoracic dysplasia 9 with or without polydactyly	*614620	9742	CL/P	CP [MGI]
IFT172	intraflagellar transport 172, chlamydomonas, homolog of	2p23.3	Short-rib thoracic dysplasia 10 with or without polydactyly	*607386	26160	CL/P	CP [68]
IFT80	intraflagellar transport 80	3q25.33	Short-rib thoracic dysplasia 2 with or without polydactyly	*611177	57560	CL/P	n.r.
IL17RD	interleukin 17 receptor D	3p14.3	Hypogonadotropic hypogonadism 18 with or without anosmia	*606807	54756	CP	n.r.
IL1B	interleukin 1 beta	2q14.1	Gastric cancer, hereditary diffuse	*147720	3553	CL/P	n.r.
IL1RN	interleukin 1 receptor antagonist	2q14.1	Gastric cancer, hereditary diffuse	*147679	3557	CL/P	n.r.
IMPAD1	inositol monophosphatase domain-containing protein 1	8q12.1	Chondrodysplasia with joint dislocations, GPAPP type	*614010	54928	CP, ICP	CP [69]
IRF6	Interferon regulatory factor 6	1q32.2	Popliteal pterygium syndrome<br>Van der Woude syndrome<br>Orofacial cleft 6	*607199	3664	CL/P, BU	CP [70,71]
JAG2	jagged2	14q32.33	-	*602570	3714	CL/P [72]	CP [73]
KANSL1	KAT8 regulatory NSL complex, subunit 1	17q21.31	Koolen-de Vries syndrome	*612452	284058	CL/P	n.r.
KAT6B	K(lysine) acetyltransferase 6B	10q22.2	Genitopatellar syndrome	*605880	23522	CP	n.r.
KCNJ2	potassium channel, inwardly rectifying, subfamily J, member 2	17q24.3	Andersen cardiodysrhythmic periodic paralysis<br>Atrial fibrillation, familial, 9	*600681	3759	CP	CP [74]
KCNQ1OT1	KCNQ1 opposite strand/antisense transcript 1 (non-protein coding)	11p15.5	Beckwith-Wiedemann syndrome	*604115	10984	CP	n.r.
KIAA0196	KIAA0196	8q24.13	Ritscher-Schinzel syndrome	*610657	9897	CP, BU	n.r.
KIF1BP /KIAA1279	KIF1 binding protein	10q22.1	Goldberg-Shprintzen syndrome	*609367	26128	CP, SCP	n.r.
KIF7	kinesin family member 7	15q26.1	Acrocallosal syndrome<br>Hydrolethalus syndrome 2	*611254	374654	CL/P	CL/P [MGI]
KISS1	KiSS-1 metastasis-suppressor	1q32.1	Hypogonadotropic hypogonadism 13 with or without anosmia	*603286	3814	CP (rare)	n.r.
KISS1R	KISS1 receptor	19p13.3	Hypogonadotropic hypogonadism 8 with or without anosmia	*604161	84634	CP (rare)	n.r.
KLHL41	kelch like family member 41	2q31.1	Nemaline myopathy 9	*607701	10324	CP (less common)	n.r.
KMT2D /MLL2	lysine-specific methyltransferase 2d	12q13.12	Kabuki syndrome 1	*602113	8085	CP	n.r.
KRAS	Kirsten rat sarcoma viral oncogene homolog	12p12.1	Gastric cancer, hereditary diffuse<br>Schimmelpenning-Feuerstein-Mims syndrome	*190070	3845	CL/P	n.r.
L1CAM	L1 cell adhesion molecule	Xq28	Hydrocephalus due to congenital stenosis of aqueduct of sylvius	*308840	3897	CP	n.r.
LARGE (?)	like-glycosyltransferase	22q12.3	Walker-Warburg Syndrome [48]	*603590	9215	CL/P (rare)	n.r.
LHX8	LIM homeobox gene 8	1p31.1	-	*604425	431707	CL/P [37]	CP [75]
LMNA	lamin A/C	1q22	Restrictive dermopathy, lethal	*150330	4000	CP, SCP	n.r.
LMX1B	LIM homeobox transcription factor 1, beta	9q33.3	Nail-Patella syndrome	*602575	4010	CL/P	n.r.
LOXL3	lysyl oxidase like 3	2p13.1	Stickler syndrome [76]	*607163	84695	CP	n.r.
LRP4	LDL receptor related protein	11p11.2	Cenani-Lenz syndactyly syndrome	*604270	4038	CP (?)	n.r.
MAFB	v-maf musculoaponeurotic fibrosarcoma oncogene family, protein B	20q12	-	*608968	9935	CL/P [1]	n.r.
MASP1	mannan-binding lectin serine peptidase 1 (C4/C2 activating component of Ra-reactive factor)	3q27.3	3MC syndrome 1	*600521	5648	CL/P	n.r.
MBTPS2	membrane-bound transcription	Xp22.12-p2	IFAP syndrome with or	*300294	51360	CP	n.r.

	factor protease, site 2	2.11	without Bresheck syndrome				
MED12	mediator complex subunit 12	Xq13.1	Lujan-Fryns syndrome Opitz-KAVEGGIA syndrome	*300188	9968	CL/P, SCP	n.r.
MEGF10	multiple epidermal growth factor-like domains 10	5q23.2	Myopathy, areflexia, respiratory distress, and dysphagia, early-onset	*612453	84466	CP	n.r.
MEOX1	mesenchyme homeobox 1	17q21.31	Klippel-feil syndrome 2, autosomal recessive	*600147	4222	CL/P	n.r.
METTL23	methyltransferase-like 23	17q25.1	Mental retardation, autosomal recessive 44	*615262	124512	CP, BU	n.r.
MID1	midline 1	Xp22.2	Opitz GBBB Syndrome, X-Linked	*300552	4281	CL/P	n.r.
MKS1	MKS1 gene	17q22	Meckel syndrome 1	*609883	54903	CL/P	CL [77]
MKX	mohawk homeobox	10p12.1	-	*601332	283078	CP [78]	n.r.
MMP9 (?)	matrix metallopeptidase 9	20q13.12	-	*120361	4318	CL/P (less common) [79]	n.r.
MSX1	muscle segment homeobox, drosophila, homolog of, 1	4p16.2	Orofacial Cleft 5 Tooth agenesis, selective, 1	*142983	4487	CL/P	CP [80,81]
MSX2	msh homeobox 2	5q35.2	Craniosynostosis, type 2 Parietal foramina 1 Parietal foramina with cleidocranial dysplasia	*123101	4488	CL/P	CP [82,83], facial cleft
MTHFD1	methylenetetrahydrofolate dehydrogenase (NADP+ dependent) 1, methenyltetrahydrofolate cyclohydrolase, formyltetrahydrofolate synthetase	14q23.3	-	*172460	4522	CL/P [84]	n.r.
MTHFR	5,10-methylenetetrahydrofolate reductase	1p36.22	Orofacial Cleft 1	*607093	4524	CL/P	n.r.
MTR	5-methyltetrahydrofolate-homocysteine S-methyltransferase	1q43	Orofacial Cleft 5	*156570	4548	CL/P	n.r.
MYH3	myosin, heavy chain 3, skeletal muscle, embryonic	17p13.1	Arthrogryposis, distal, type 2B	*160720	4621	CL/P	n.r.
MYH9	myosin, heavy chain 9, nonmuscle	22q12.3	-	*160775	4627	CL/P [85]	n.r.
NAT1 (?)	N-acetyltransferase 1	8p22	-	*108345	9	CL/P [86]	CL/P [87]
NAT2 (?)	N-acetyltransferase 2	8p22	-	*612182	10	CL/P [86]	n.r.
NBN	nibrin	8q21.3	Nijmegen breakage syndrome	*602667	4683	CL/P	n.r.
NEB	nebulin	2q23.3	Nemaline myopathy 2	*161650	4703	CP	n.r.
NEK1	NIMA related kinase 1	4q33	Short-rib thoracic dysplasia 6 with or without polydactyly	*604588	4750	CL/P	n.r.
NIPBL	nipped-B-like	5p13.2	Cornelia de Lange syndrome 1	*608667	25836	CL/P	n.r.
NKX2-5	NK2 homeobox 5	5q35.1	Conotruncal heart malformations	*600584	1482	CP, SCP, BU	n.r.
NKX2-6	NK2 homeobox 6	8p21.2	Conotruncal heart malformations	*611770	137814	CP, SCP, BU	n.r.
NOG	noggin	17q22	-	*602991	9241	CL/P [88]	n.r.
NOS3	nitric oxide synthase 3	7q36.1	-	+163729	4846	CL/P [89]	n.r.
NRAS	neuroblastoma RAS viral (v-ras) oncogene homolog	1p13.2	Schimmelpenning-Feuerstein-Mims syndrome	*164790	4893	CP	n.r.
NSD1	nuclear receptor binding SET domain protein 1	5q35.2	Beckwith-Wiedemann syndrome	*606681	64324	CP	n.r.
NSDHL	NAD(P) dependent steroid dehydrogenase-like	Xq28	CHILD syndrome	*300275	50814	CL	n.r.
NSMF	NMDA receptor synaptonuclear signaling and neuronal migration factor	9q34.3	Hypogonadotropic hypogonadism 9 with or without anosmia	*608137	26012	CP	n.r.
NTN1	netrin 1, mouse, homolog of	17p13.1	-	*601614	9423	CL/P [90]	n.r.
OFCC1	OFC1 candidate gene 1	6p24.3	Orofacial Cleft 1	*614287	266553	CL/P	n.r.
OFD1	OFD1 gene	Xp22.2	Orofaciodigital syndrome I Simpson-Golabi-Behmel syndrome, type 2	*300170	8481	CL/P, BU	CP [91]
ORC1	origin recognition complex, subunit 1	1p32.3	Meier-Gorlin syndrome 1	*601902	4998	CP, BU	n.r.
OTX2	orthodenticle homeobox 2	14q22.3	Microphthalmia, syndromic 5	*600037	5015	CP (less common)	n.r.
PAFAH1B1	platelet activating factor acetylhydrolase 1b regulatory subunit 1	17p13.3	17p13.3 microduplication syndrome	*601545	5048	CL/P [92]	n.r.
PAX3	paired box gene 3	2q36.1	Waardenburg syndrome, type 1 Waardenburg syndrome, type 3	*606597	5077	CL/P	n.r.
PAX7	paired box gene 7	1p36.13	-	* 167410	5081	CL/P [90]	n.r.
PDGFC	platelet-derived growth factor C	56034	-	*608452	56034	CL/P [93]	CP [94]

PDGFRA	platelet-derived growth factor receptor, alpha	4q12	-	*173490	5156	CP [95]	CP [96,97]
PEX5	peroxisomal biogenesis factor 5	12p13.31	Peroxisome biogenesis disorder 2a	*600414	5830	CP	n.r.
PEX7	peroxisomal biogenesis factor 7	6q23.3	Rhizomelic chondrodysplasia punctata, type 1	*601757	5191	CP	n.r.
PGAP2	post-GPI attachment to proteins 2	11p15.4	Hyperphosphatasia with mental retardation syndrome 3	*615187	27315	CP (rare)	n.r.
PGAP3	post-GPI attachment to proteins 3	17q12	Hyperphosphatasia with mental retardation syndrome 4	*611801	93210	CP (less common)	n.r.
PGM1	phosphoglucomutase 1	1p31.3	Congenital disorder of glycosylation, type It	*171900	5236	CP, BU	n.r.
PHF8	PHD finger protein 8	Xp11.22	Siderius X-linked mental retardation syndrome	*300560	23133	CL/P	n.r.
PHGDH	phosphoglycerate dehydrogenase	1p12	Neu-Laxova syndrome 1	*606879	26227	CL/P	n.r.
PIEZO2	piezo-type mechanosensitive ion channel component 2	18p11.22-p11.21	Arthrogryposis, distal, type 3<br>Arthrogryposis, distal, type 5<br>Marden-Walker syndrome	*613629	63895	CP, BU, SCP	n.r.
PIGL	phosphatidylinositol glycan anchor biosynthesis, class L	17p11.2	Coloboma, congenital heart disease, ichthyosiform dermatosis, mental retardation, and ear anomalies syndrome	*605947	9487	CP, BU	n.r.
PIGV	phosphatidylinositol glycan, class V	1p36.11	Hyperphosphatasia with mental retardation syndrome 1	*610274	55650	CL/P, CP (rare)	CP [MGI]
PIK3R2	phosphoinositide-3-kinase, regulatory subunit 2 (beta)	19p13.11	Megalencephaly-polymicrogyria-polydactyly-hydrocephalus syndrome 1	*603157	5296	CP, SCP	n.r.
PITX1	paired-like homeodomain transcription factor 1	5q31.1	Clubfoot, congenital, with or without deficiency of long bones and/or mirror-image polydactyly	*602149	5307	CP	CP [98,99]
PLCB4	phospholipase C, beta-4	20p12.3-p12.2	Auriculocondylar syndrome 2	*600810	5332	CP	n.r.
POLR1C	polymerase (RNA) I polypeptide C	6p21.1	Treacher Collins syndrome 3	*610060	9533	CP	n.r.
POLR1D	polymerase (RNA) I polypeptide D	13q12.2	Treacher Collins syndrome 2	*613715	51082	CP	n.r.
POMT1	protein-O-mannosyltransferase 1	9q34.13	Muscular dystrophy-dystroglycanopathy (congenital with brain and eye anomalies)	*607423	10585	CL/P	n.r.
POMT2	protein-O-mannosyltransferase 2	14q24.3	Muscular dystrophy-dystroglycanopathy (congenital with brain and eye anomalies), type A, 2	*607439	29954	CL/P	n.r.
PORCN	porcupine, drosophila, homolog of	Xp11.23	Focal dermal hypoplasia	*300651	64840	CL/P	CP [100]
PQBP1	polyglutamine-binding protein 1	Xp11.23	Renpenning syndrome 1	*300463	10084	CP	n.r.
PRBNS	Pierre Robin syndrome	17q24.3-q25.1	Pierre Robin syndrome	%261800	100301572	CP	n.r.
PROKR2	prokineticin receptor 2	20p12.3	Hypogonadotropic hypogonadism 3 with or without anosmia	*607123	128674	CP (rare)	n.r.
PRRX1	paired related homeobox 1	1q24.2	Agnathia-otocephaly complex	*167420	5396	CP	CP [101-103]
PSAT1	phosphoserine aminotransferase 1	9q21.2	Neu-Laxova syndrome 2	*610936	29968	CP	n.r.
PTCH1	patched, drosophila, homolog of, 1	9q22.32	Holoprosencephaly 7<br>Basal cell nevus syndrome	*601309	5727	CL/P	CP [104]
PTCH2	patched, drosophila, homolog of, 2	1p34.1	Basal cell nevus syndrome	*603673	8643	CL/P	n.r.
PTDSS1	phosphatidylserine synthase 1	8q22.1	Lenz-Majewski hyperostotic dwarfism	*612792	9791	CP	n.r.
PTEN (?)	phosphatase and tensin homolog	10q23.31	VATER association with macrocephaly and ventriculomegaly	+601728	5728	CP	n.r.
PTPN11	protein tyrosine phosphatase, non-receptor type 11	12q24.13	LEOPARD syndrome 1	*176876	5781	CP	n.r.
PVRL1	poliovirus receptor-like 1; nectin-1	11q23.3	Cleft lip/palate-ectodermal dysplasia syndrome	*600644	5818	CL/P	n.r.
RAD21	RAD21 cohesin complex component	8q24.11	Cornelia de Lange syndrome 4	*606462	5885	CP, SCP	n.r.
RAI1	retinoic acid induced 1	17p11.2	Smith-Magenis syndrome	*607642	10743	CP	n.r.
RAPSN	receptor associated protein of the synapse	11p11.2	Fetal akinesia deformation sequence	*601592	5913	CP	n.r.
RARA	retinoic acid receptor alpha	17q21.2	-	*180240	5914	CL/P [105]	n.r.
RARB	retinoic acid receptor beta	3p24.2	Microphthalmia, syndromic 12	*180220	5915	CP (rare)	n.r.
RB1	retinoblastoma 1	13q14.2	Retinoblastoma	*614041	5925	CP (rare)	n.r.
RBM10	RNA-binding motif protein 10	Xp11.23	Tarp syndrome	*300080	8241	CP	n.r.
RBM8A	RNA binding motif protein 8A	1q21.1	Thrombocytopenia-absent radius syndrome	*605313	9939	CL/P (rare), IPC (rare)	n.r.
RECQL4	RecQ protein-like 4	8q24.3	Baller-Gerold syndrome<br>RAPADILINO syndrome	*603780	9401	CP	CP [106]

			Rothmund-Thomson syndrome				
RIPK4	receptor interacting serine/threonine kinase 4	21q22.3	Popliteal pterygium syndrome, lethal type	*605706	54101	CL/P	n.r.
ROR2	receptor tyrosine kinase-like orphan receptor 2	9q22.31	Robinow syndrome, autosomal recessive	*602337	4920	CL/P	CP [107]
RPGRIP1L	RPGRIP1-like	16q12.2	Meckel syndrome, type 5	*610937	23322	CL/P	n.r.
RPL11	ribosomal protein L11	1p36.11	Diamond-Blackfan anemia 7	*604175	6135	CL/P	n.r.
RPL26	ribosomal protein L26	17p13.1	?Diamond-Blackfan anemia 11	*603704	6154	CL/P	n.r.
RPL5	ribosomal protein L5	1p22.1	Diamond-Blackfan anemia 6	*603634	6125	CL/P, ICP, BU	n.r.
RPS17	ribosomal protein S17	15q25.2	Diamond-Blackfan anemia 4	*180472	6218	CL/P	n.r.
RPS19	ribosomal protein S19	19q13.2	Diamond-Blackfan anemia 1	*603474	6223	CL/P	n.r.
RPS26	ribosomal protein S26	12q13.2	Diamond-Blackfan anemia 10	*603701	6231	CL/P	n.r.
RPS7 (?)	ribosomal protein S7	2p25.3	Diamond-Blackfan anemia 8	*603658	6201	CL/P	n.r.
RUNX2	runt-related transcription factor 2	6p21.1	Cleidocranial dysplasia	*600211	860	CP, SCP	CP [108]
RYK	receptor-like tyrosine kinase	3q22.2	-	*600524	6259	CL/P [109]	CP [110]
SATB2 (#)	special AT-rich sequence-binding protein 2	2q33.1	Glass syndrome	*608148	23314	CP, ICP	CL/P [111,112]
SCARF2	scavenger receptor class F member 2	22q11.21	Van den Ende-Gupta syndrome	*613619	91179	CP	n.r.
SCD5	stearoyl-CoA desaturase 5	4q21.22	-	*608370	79966	CL	n.r.
SCLT1	sodium channel and clathrin linker 1	4q28.2	-	*611399	132320	CL/P [113]	n.r.
SEC23A	Sec23 homolog A, COPII coat complex component	14q21.1	Craniolenticulosutural dysplasia	*610511	10484	CP, BU	n.r.
SEMA3E	semaphorin 3E	7q21.11	CHARGE syndrome	*608166	9723	CL/P	n.r.
SEPT9	septin 9	17q25.2-q25.3	Amyotrophy, hereditary neuralgic	*604061	10801	CP	n.r.
SF3B4	splicing factor 3b subunit 4	1q21.2	Acrofacial dysostosis 1, Nager type	*605593	10262	CL/P	n.r.
SHH	sonic hedgehog	7q36.3	Holoprosencephaly 3	*600725	6469	CL/P	CP [40,114]
SIX3	SIX homeobox 3	2p21	Holoprosencephaly 2	*603714	6496	CL/P, anterior CP, BU, SCP	n.r.
SKI	SKI proto-oncogene	1p36.33-p36.32	Shprintzen-Goldberg syndrome	*164780	6497	CL/P (rare) [72]	n.r.
SLC16A1	solute carrier family 16 (monocarboxylic acid transporter), member 1	1p13.2	-	*600682	6566	CP [115]	n.r.
SLC26A2	solute carrier family 26 (sulfate transporter), member 2	5q32	Atelosteogenesis, type II<br>Diastrophic dysplasia<br>Epiphyseal dysplasia, multiple, 4	*606718	1836	CP	n.r.
SLC35D1	solute carrier family 35 (udp-glucuronic acid/udp-n-acetylgalactosamine dual transporter), member d1	1p31.3	Schneckenbecken dysplasia	*610804	23169	CP	n.r.
SLC39A13	solute carrier family 39 (zinc transporter), member 13	11p11.2	Spondylocheirodysplasia, Ehlers-Danlos syndrome-like	*608735	91252	BU	n.r.
SMAD3	mothers against decapentaplegic, drosophila, homolog of, 3	15q22.33	Loeys-Dietz syndrome 3	*603109	4088	CP, BU	n.r.
SMAD4 /MADH4	SMAD family member 4	18q21.2	Myhre syndrome	*600993	4089	CL/P (less common)	CP [116]
SMC1A /DXS423E	structural maintenance of chromosomes 1A	Xp11.22	Cornelia de Lange syndrome 2	*300040	8243	CP (rare)	n.r.
SMOC1	SPARC related modular calcium binding 1	14q24.2	Microphthalmia with limb anomalies	*608488	64093	CL/P	CP [117]
SMS	spermine synthase	Xp22.11	Mental retardation, X-linked, syndromic, Snyder-Robinson type	*300105	6611	CP, BU	n.r.
SNRPB	small nuclear ribonucleoprotein polypeptides B and B1	20p13	Cerebrocostomandibular syndrome	*182282	6628	CP, ICP	n.r.
SNX3 (?)	sorting nexin 3	6q21	-	*605930	8724	CL/P [118]	n.r.
SOX2	SRY-box 2	3q26.33	Microphthalmia, syndromic 3	*184429	6657	CP	n.r.
SOX9	SRY-box 9	17q24.3	Campomelic dysplasia	*608160	6662	CP	CP [119,120]
SPECC1L	sperm antigen with calponin homology and coiled-coil domains 1-like	22q11.23	Facial clefting, oblique, 1<br>Opitz GBBB syndrome, type II	*614140	23384	CL/P	n.r.
SPEG	SPEG complex locus	2q35	Myopathy, centronuclear, 5	*615950	10290	BU	n.r.
SPINT2	serine peptidase inhibitor, Kunitz type, 2	19q13.2	Diarrhea 3, secretory sodium, congenital, with or without other congenital anomalies	*605124	10653	CP, BU	n.r.
SPRY2	sprouty homolog 2	13q31.1	-	*602466	10253	CL/P [88]	CP [121,122]
SPRY4 (?)	sprouty homolog 4	5q31.3	Hypogonadotropic hypogonadism 17 with or without anosmia	*607984	81848	CP (rare)	n.r.

SRY	sex determining region Y	Yp11.31	46,XY sex reversal 1	*480000	6736	CP	n.r.
ST5	suppressor of tumorigenicity 5	11p15.4	-	*140750	6764	CP [123]	n.r.
STAC3	SH3 and cysteine rich domain 3	12q13.3	Native American myopathy	*615521	246329	CP	n.r.
STAMBP	STAM binding protein	2p13.1	Microcephaly-capillary malformation syndrome	*606247	10617	CP	n.r.
STRA6	stimulated by retinoic acid 6	15q24.1	Microphthalmia, syndromic 9	*610745	64220	CP	n.r.
STXBP1	syntaxin binding protein 1	9q34.11	Epileptic encephalopathy, early infantile, 4	*602926	6812	CL/P	n.r.
SUFU	SUFU negative regulator of hedgehog signaling	10q24.32	Basal cell nevus syndrome<br>Medulloblastoma, desmoplastic	*607035	51684	CL/P	CP [MGI]
SUMO1	small ubiquitin-like modifier 1	2q33.1	Orofacial Cleft 10	*601912	7341	CL/P	CP [124]
TAC3	tachykinin 3	12q13.3	Hypogonadotropic hypogonadism 10 with or without anosmia	*162330	6866	CP (rare)	n.r.
TACR3	tachykinin receptor 3	4q24	Hypogonadotropic hypogonadism 11 with or without anosmia	*162332	6870	CP (rare)	n.r.
TBC1D32	TBC1 domain family, member 32	6q22.31	Oro-Facio-Digital syndrome type IX	*615867	221322	CL/P	CL/P [MGI]
TBCE	tubulin folding cofactor E	1q42.3	Hypoparathyroidism-retardatio n -dysmorphism syndrome	*604934	6905	BU	n.r.
TBX1	T-box 1	22q11.21	Conotruncal heart malformations<br>Digeorge syndrome<br>Velocardiofacial syndrome	*602054	6899	CP, BU, SCP	CP [125,126]
TBX10	T-box 10	11q13.2	-	*604648	347853	CL/P [72]	CL/P [127]
TBX15	T-box 15	1p12	Cousin syndrome	*604127	6913	CP	n.r.
TBX22	T-box 22	Xq21.1	Abruzzo-Erickson syndrome<br>Cleft palate with or without ankyloglossia, XL	*300307	50945	CP, BU, IPC, SCP	CP [128]
TBX4	T-box 4	17q23.2	Ischiocoxopodopatellar syndrome	*601719	9496	CP	n.r.
TCN2	transcobalamin 2	22q12.2	-	*613441	6948	CL/P [129]	n.r.
TCOF1	Treacher Collins-Franceschetti syndrome 1	5q32-q33	Treacher Collins syndrome 1	*606847	6949	CP	CP [130]
TCTN2	tectonic family member 2	12q24.31	Meckel syndrome 8<br>Joubert syndrome 24	*613846	79867	CL/P	CP [131]
TCTN3	tectonic family member 3	10q24.1	Orofaciodigital syndrome IV	*613847	26123	CP	n.r.
TFAP2A	transcription factor AP2-alpha	6p24.3	Branchiooculofacial syndrome	*107580	7020	CL/P	CL/P [132]
TGDS	TDP-glucose 4,6-dehydratase	13q32.1	Catel-manzke syndrome	*616146	23483	CL/P	n.r.
TGFA	transforming growth factor, alpha	2p13.3	Orofacial cleft 2	*190170	7039	CL/P	n.r.
TGFB2	transforming growth factor, beta-2	1q41	Loeys-Dietz syndrome 4	*190220	7042	CP, BU	CP [133]
TGFB3	transforming growth factor, beta-3	14q24.3	Arrhythmogenic right ventricular dysplasia 1<br>Loeys-Dietz syndrome 5	*190230	7043	CP, ICP, BU	CP [134-136]
TGFBR1	transforming growth factor-beta receptor, type I	9q22.33	Loeys-Dietz syndrome 1	*190181	7046	CP, BU	CP [137,138]
TGFBR2	transforming growth factor-beta receptor, type II	3p24.1	Loeys-Dietz syndrome 2	*190182	7048	CP, BU	CP [139,140]
TGIF1	transforming growth factor-beta-induced factor homeobox 1	18p11.31	Holoprosencephaly 4	*602630	7050	CL/P	n.r.
TMCO1	transmembrane and coiled-coil domains 1	1q24.1	Craniofacial dysmorphism, skeletal anomalies, and mental retardation syndrome	*614123	54499	CL/P	n.r.
TMEM216	transmembrane protein 216	11q12.2	Meckel syndrome, type 2	*613277	51259	CP	n.r.
TMEM67	transmembrane protein 67	8q22.1	Meckel syndrome, type 3	*609884	91147	CP	n.r.
TNNI2	troponin I type 2	11p15.5	Arthrogryposis, distal, type 2B	*191043	7136	CL/P	n.r.
TNNT3	troponin T type 3	11p15.5	Arthrogryposis, distal, type 2B	*600692	7140	CL/P	n.r.
TNXB	tenascin XB	6p21.33	Ehlers-Danlos-like syndrome due to tenascin-X deficiency	*600985	7148	BU	n.r.
TP63	tumor protein p63	3q28	ADULT syndrome<br>Ectrodactyly, ectodermal dysplasia, and cleft lip/palate syndrome<br>Hay-Wells syndrome<br>Limb-mammary syndrome<br>Orofacial cleft 8<br>Rapp-Hodgkin syndrome<br>Split-hand/foot malformation 4	*603273	8626	CL/P, BU	CP [141]
TPM2	tropomyosin 2 (beta)	9p13.3	Arthrogryposis, distal, type 2B	*190990	7169	CL/P	n.r.
TTC21B	tetratricopeptide repeat domain 21B	2q24.3	Short-rib thoracic dysplasia 4 with or without polydactyly	*612014	79809	CL/P	n.r.
TTC37	tetratricopeptide repeat domain 37	5q15	Trichohepatoenteric syndrome	*614589	9652	BU	n.r.

TWIST1	twist, drosophila, homolog of, 1	7p21.1	Craniosynostosis, type 1<br>Robinow-Sorauf syndrome<br>Saethre-Chotzen syndrome	*601622	7291	CP	CP [142]
TXNL4A	thioredoxin-like 4A	18q23	Burn-Mckeown syndrome	*611595	10907	CL/P, BU	n.r.
UBB	ubiquitin B	17p11.2	Cleft palate, isolated, and mental retardation	*191339	7314	CP, ICP	n.r.
UFD1L (?)	ubiquitin fusion degradation 1-like	22q11.21	-	*601754	7353	CP [143]	n.r.
UQCC2	ubiquinol-cytochrome c reductase complex assembly factor 2	6p21.31	Mitochondrial complex III deficiency, nuclear type 7	*614461	84300	CP (rare)	n.r.
VAX1	ventral anterior homeobox 1	10q25.3	Microphthalmia, syndromic 11	*604294	11023	CL/P	CP [144]
VWSM	van der Woude syndrome modifier	17p11.2	Van der Woude syndrome-1	%604547	100917	CL/P	n.r.
WDR11	WD repeat domain 11	10q26.12	Hypogonadotropic hypogonadism 14 with or without anosmia	*606417	55717	CP (rare)	n.r.
WDR19	WD repeat domain 19	4p14	Cranioectodermal dysplasia 4<br>Short-rib thoracic dysplasia 5 with or without polydactyly	*608151	57728	CL/P	CL/P [145]
WDR34	WD repeat domain 34	9q34.11	Short-rib thoracic dysplasia 11 with or without polydactyly	*613363	89891	CL/P	n.r.
WDR35	WD repeat domain 35	2p24.1	Short-rib thoracic dysplasia 7 with or without polydactyly	*613602	57539	CL/P	n.r.
WDR60	WD repeat domain 60	7q36.3	Short-rib thoracic dysplasia 8 with or without polydactyly	*615462	55112	CL/P	n.r.
WHSC1	Wolf-Hirschhorn syndrome candidate 1	4p16.3	Wolf-Hirschhorn syndrome candidate 1	*602952	7468	CL/P	CP [146]
WNT3	wingless-type MMTV integration site family, member 3	17q21.31	Tetraamelia syndrome, autosomal recessive	*165330	7473	CL/P	n.r.
WNT5A	wingless-type MMTV integration site family, member 5A	3p14.3	Robinow syndrome, autosomal dominant 1	*164975	7474	CL/P	CL/P [147,148]
WNT7A	wingless-type MMTV integration site family, member 7A	3p25.1	Fuhrmann syndrome<br>Ulna and fibula, absence of, with severe limb deficiency	*601570	7476	CL/P	n.r.
WT1	Wilms tumor 1	11p13	Denys-Drash syndrome	*607102	7490	CP	n.r.
XYLT1	xylosyltransferase I	16p12.3	Desbuquois dysplasia 2	*608124	64131	CP (less common)	n.r.
YAP1	Yes-associated protein 1, 65-KD	11q22.1	Coloboma, ocular, with or without hearing impairment, cleft lip/palate, and/or mental retardation	*606608	10413	CL/P	n.r.
YWHAE (?)	tyrosine 3-monooxygenase/tryptophan 5-monooxygenase activation protein, epsilon	17p13.3	17p13.3 microduplication syndrome	*605066	7531	CL/P [92]	n.r.
ZEB2	zinc finger E box-binding homeobox 2	2q22.3	Mowat-Wilson syndrome	*605802	9839	CP, SCP	n.r.
ZIC2	zinc finger protein of the cerebellum 2	20q13.2	Holoprosencephaly 5	*603073	7546	CL/P	n.r.
ZIC3	zinc finger protein of the cerebellum 3	Xq26.3	Congenital heart defects, nonsyndromic<br>Heterotaxy, visceral, 1<br>VACTERL association, X-linked	*300265	7547	CP	CP [149]
ZMPSTE24	zinc metallopeptidase STE24	1p34.2	Restrictive dermopathy, lethal	*606480	10269	CP, SCP	n.r.
ZSWIM6	zinc finger, SWIM-type containing 6	5q12.1	Acromelic frontonasal dysostosis	*615951	57688	CL/P	n.r.

Ref., references; OMIM, Online Mendelian Inheritance in Man (http://omim.org); CP, cleft palate; CL, cleft lip; CL/P, cleft lip and/or palate; CLP, cleft lip and palate; ICP, incomplete cleft palate; BU, bifid uvula; n.r., not reported; MGI, Mouse Genome Informatics (MGI; http://www.informatics.jax.org/) direct data submission.
An asterisk (*) before an OMIM entry number indicates a gene.
A plus sign (+) before an OMIM entry number indicates that the entry includes a description of a gene and a phenotype.
A percentage sign (%) before an OMIM entry number indicates that the entry describes a confirmed Mendelian phenotype or phenotypic locus for which the underlying molecular basis is not known.
A hash sign (#) after gene indicates that the CP phenotype could be induced by a secondary defect.
A question mark, "?" indicates an unconfirmed mapping.

Supplemental References

1. Beaty TH, Murray JC, Marazita ML*, et al.* A genome-wide association study of cleft lip with and without cleft palate identifies risk variants near MAFB and ABCA4. *Nat Genet* 2010;42:525-529.
2. Bliek BJ, van Schaik RH, van der Heiden IP*, et al.* Maternal medication use, carriership of the ABCB1 3435C > T polymorphism and the risk of a child with cleft lip with or without cleft palate. *Am J Med Genet A* 2009;149A:2088-2092.
3. Wolf ZT, Brand HA, Shaffer JR*, et al.* Genome-wide association studies in dogs and humans identify ADAMTS20 as a risk variant for cleft lip and palate. *PLoS Genet* 2015;11:e1005059.
4. Enomoto H, Nelson CM, Somerville RP*, et al.* Cooperation of two ADAMTS metalloproteases in closure of the mouse palate identifies a requirement for versican proteolysis in regulating palatal mesenchyme proliferation. *Development* 2010;137:4029-4038.
5. Uz E, Alanay Y, Aktas D*, et al.* Disruption of ALX1 causes extreme microphthalmia and severe facial clefting: expanding the spectrum of autosomal-recessive ALX-related frontonasal dysplasia. *Am J Hum Genet* 2010;86:789-796.
6. Beverdam A, Brouwer A, Reijnen M*, et al.* Severe nasal clefting and abnormal embryonic apoptosis in Alx3/Alx4 double mutant mice. *Development* 2001;128:3975-3986

7. Kayano S, Suzuki Y, Kanno K, *et al.* Significant association between nonsyndromic oral clefts and arylhydrocarbon receptor nuclear translocator (ARNT). *Am J Med Genet A* 2004;130A:40-44.
8. Grubben C, Fryns JP, De Zegher F, *et al.* Anterior basal encephalocele in the median cleft face syndrome. Comments on nosology and treatment. *Genet Couns* 1990;1:103-109.
9. Abdel-Wahab O, Gao J, Adli M, *et al.* Deletion of Asxl1 results in myelodysplasia and severe developmental defects in vivo. *J Exp Med* 2013;210:2641-2659.
10. Stein J, Mulliken JB, Stal S, *et al.* Nonsyndromic cleft lip with or without cleft palate: evidence of linkage to BCL3 in 17 multigenerational families. *Am J Hum Genet* 1995;57:257-272.
11. Bonilla-Claudio M, Wang J, Bai Y, *et al.* Bmp signaling regulates a dose-dependent transcriptional program to control facial skeletal development. *Development* 2012;139:709-719.
12. Matzuk MM, Kumar TR, Bradley A. Different phenotypes for mice deficient in either activins or activin receptor type II. *Nature* 1995;374:356-360.
13. Matzuk MM, Kumar TR, Vassalli A, *et al.* Functional analysis of activins during mammalian development. *Nature* 1995;374:354-356.
14. Atasoy D, Schoch S, Ho A, *et al.* Deletion of CASK in mice is lethal and impairs synaptic function. *Proc Natl Acad Sci U S A* 2007;104:2525-2530
15. Wilson JB, Ferguson MW, Jenkins NA, *et al.* Transgenic mouse model of X-linked cleft palate. *Cell Growth Differ* 1993;4:67-76.
16. Yan Y, Frisen J, Lee MH, *et al.* Ablation of the CDK inhibitor p57Kip2 results in increased apoptosis and delayed differentiation during mouse development. *Genes Dev* 1997;11:973-983.
17. Zhang P, Liegeois NJ, Wong C, *et al.* Altered cell differentiation and proliferation in mice lacking p57KIP2 indicates a role in Beckwith-Wiedemann syndrome. *Nature* 1997;387:151-158.
18. Zhang W, Hong M, Bae GU, *et al.* Boc modifies the holoprosencephaly spectrum of Cdo mutant mice. *Dis Model Mech* 2011;4:368-380.
19. Bosman EA, Penn AC, Ambrose JC, *et al.* Multiple mutations in mouse Chd7 provide models for CHARGE syndrome. *Hum Mol Genet* 2005;14:3463-3476.
20. Sperry ED, Hurd EA, Durham MA, *et al.* The chromatin remodeling protein CHD7, mutated in CHARGE syndrome, is necessary for proper craniofacial and tracheal development. *Dev Dyn* 2014;243:1055-1066.
21. Turhani D, Item CB, Watzinger E, *et al.* Mutation analysis of CLPTM 1 and PVRL 1 genes in patients with non-syndromic clefts of lip, alveolus and palate. *J Craniomaxillofac Surg* 2005;33:301-306.
22. Seegmiller R, Fraser FC, Sheldon H. A new chondrodystrophic mutant in mice. Electron microscopy of normal and abnormal chondrogenesis. *J Cell Biol* 1971;48:580-593.
23. Li SW, Prockop DJ, Helminen H, *et al.* Transgenic mice with targeted inactivation of the Col2 alpha 1 gene for collagen II develop a skeleton with membranous and periosteal bone but no endochondral bone. *Genes Dev* 1995;9:2821-2830.
24. Beaty TH, Taub MA, Scott AF, *et al.* Confirming genes influencing risk to cleft lip with/without cleft palate in a case-parent trio study. *Hum Genet* 2013;132:771-781.
25. Lachman HM, Morrow B, Shprintzen R, *et al.* Association of codon 108/158 catechol-O-methyltransferase gene polymorphism with the psychiatric manifestations of velo-cardio-facial syndrome. *Am J Med Genet* 1996;67:468-472.
26. Chiquet BT, Lidral AC, Stal S, *et al.* CRISPLD2: a novel NSCLP candidate gene. *Hum Mol Genet* 2007;16:2241-2248.
27. Wassif CA, Zhu P, Kratz L, *et al.* Biochemical, phenotypic and neurophysiological characterization of a genetic mouse model of RSH/Smith--Lemli--Opitz syndrome. *Hum Mol Genet* 2001;10:555-564.
28. Fitzky BU, Moebius FF, Asaoka H, *et al.* 7-Dehydrocholesterol-dependent proteolysis of HMG-CoA reductase suppresses sterol biosynthesis in a mouse model of Smith-Lemli-Opitz/RSH syndrome. *J Clin Invest* 2001;108:905-915.
29. Kantarci S, Ackerman KG, Russell MK, *et al.* Characterization of the chromosome 1q41q42.12 region, and the candidate gene DISP1, in patients with CDH. *Am J Med Genet A* 2010;152A:2493-2504.
30. Acampora D, Merlo GR, Paleari L, *et al.* Craniofacial, vestibular and bone defects in mice lacking the Distal-less-related gene Dlx5. *Development* 1999;126:3795-3809
31. Depew MJ, Liu JK, Long JE, *et al.* Dlx5 regulates regional development of the branchial arches and sensory capsules. *Development* 1999;126:3831-3846.
32. Kurihara Y, Kurihara H, Suzuki H, *et al.* Elevated blood pressure and craniofacial abnormalities in mice deficient in endothelin-1. *Nature* 1994;368:703-710.
33. Compagni A, Logan M, Klein R, *et al.* Control of skeletal patterning by ephrinB1-EphB interactions. *Dev Cell* 2003;5:217-230.
34. Bush JO, Soriano P. Ephrin-B1 regulates axon guidance by reverse signaling through a PDZ-dependent mechanism. *Genes Dev* 2009;23:1586-1599.
35. Osoegawa K, Vessere GM, Utami KH, *et al.* Identification of novel candidate genes associated with cleft lip and palate using array comparative genomic hybridisation. *J Med Genet* 2008;45:81-86.
36. Xu PX, Adams J, Peters H, *et al.* Eya1-deficient mice lack ears and kidneys and show abnormal apoptosis of organ primordia. *Nat Genet* 1999;23:113-117.
37. Nikopensius T, Kempa I, Ambrozaityte L, *et al.* Variation in FGF1, FOXE1, and TIMP2 genes is associated with nonsyndromic cleft lip with or without cleft palate. *Birth Defects Res A Clin Mol Teratol* 2011;91:218-225.
38. Wang H, Zhang T, Wu T, *et al.* The FGF and FGFR Gene Family and Risk of Cleft Lip With or Without Cleft Palate. *Cleft Palate Craniofac J* 2013;50:96-103.
39. Riley BM, Mansilla MA, Ma J, *et al.* Impaired FGF signaling contributes to cleft lip and palate. *Proc Natl Acad Sci U S A* 2007;104:4512-4517.
40. Rice R, Spencer-Dene B, Connor EC, *et al.* Disruption of Fgf10/Fgfr2b-coordinated epithelial-mesenchymal interactions causes cleft palate. *J Clin Invest* 2004;113:1692-1700.
41. Alappat SR, Zhang Z, Suzuki K, *et al.* The cellular and molecular etiology of the cleft secondary palate in Fgf10 mutant mice. *Dev Biol* 2005;277:102-113
42. Green RM, Feng W, Phang T, *et al.* Tfap2a-dependent changes in mouse facial morphology result in clefting that can be ameliorated by a reduction in Fgf8 gene dosage. *Dis Model Mech* 2015;8:31-43.
43. Trokovic N, Trokovic R, Mai P, *et al.* Fgfr1 regulates patterning of the pharyngeal region. *Genes Dev* 2003;17:141-153.
44. De Moerlooze L, Spencer-Dene B, Revest JM, *et al.* An important role for the IIIb isoform of fibroblast growth factor receptor 2 (FGFR2) in mesenchymal-epithelial signalling during mouse organogenesis. *Development* 2000;127:483-492.
45. Hosokawa R, Deng X, Takamori K, *et al.* Epithelial-specific requirement of FGFR2 signaling during tooth and palate development. *J Exp Zool B Mol Dev Evol* 2009;312B:343-350.
46. Hart AW, Morgan JE, Schneider J, *et al.* Cardiac malformations and midline skeletal defects in mice lacking filamin A. *Hum Mol Genet* 2006;15:2457-2467.
47. Vajsar J, Baskin B, Swoboda K, *et al.* Walker-Warburg Syndrome with POMT1 mutations can be associated with cleft lip and cleft palate. *Neuromuscul Disord* 2008;18:675-677.
48. van Reeuwijk J, Brunner HG, van Bokhoven H. Glyc-O-genetics of Walker-Warburg syndrome. *Clin Genet* 2005;67:281-289.
49. Iida K, Koseki H, Kakinuma H, *et al.* Essential roles of the winged helix transcription factor MFH-1 in aortic arch patterning and skeletogenesis. *Development* 1997;124:4627-4638.
50. Moreno LM, Mansilla MA, Bullard SA, *et al.* FOXE1 association with both isolated cleft lip with or without cleft palate, and isolated cleft palate. *Hum Mol Genet* 2009;18:4879-4896.
51. De Felice M, Ovitt C, Biffali E, *et al.* A mouse model for hereditary thyroid dysgenesis and cleft palate. *Nat Genet* 1998;19:395-398.
52. Caruana G, Farlie PG, Hart AH, *et al.* Genome-wide ENU mutagenesis in combination with high density SNP analysis and exome sequencing provides rapid identification of novel mouse models of developmental disease. *PLoS One* 2013;8:e55429.
53. Li P, Zhang HZ, Huff S, *et al.* Karyotype-phenotype insights from 11q14.1-q23.2 interstitial deletions: FZD4 haploinsufficiency and exudative vitreoretinopathy in a patient with a complex chromosome rearrangement. *Am J Med Genet A* 2006;140:2721-2729.
54. Vieira AR, Howe A, Murray JC. Studies of gamma-aminobutyric acid type A receptor beta3 (GABRB3) and glutamic acid decarboxylase 67 (GAD67) with oral clefts. *Am J Med Genet A* 2008;146A:2828-2830.
55. Filezio MR, Bagordakis E, de Aquino SN, *et al.* Polymorphisms in GABRB3 and oral clefting in the Brazilian population. *DNA Cell Biol* 2013;32:125-129.
56. Scapoli L, Martinelli M, Pezzetti F, *et al.* Linkage disequilibrium between GABRB3 gene and nonsyndromic familial cleft lip with or without cleft palate. *Hum Genet* 2002;110:15-20.
57. Homanics GE, DeLorey TM, Firestone LL, *et al.* Mice devoid of gamma-aminobutyrate type A receptor beta3 subunit have epilepsy, cleft palate, and hypersensitive behavior. *Proc Natl Acad Sci U S A* 1997;94:4143-4148.

58. Ribeiro LA, Quiezi RG, Nascimento A, *et al.* Holoprosencephaly and holoprosencephaly-like phenotype and GAS1 DNA sequence changes: Report of four Brazilian patients. *Am J Med Genet A* 2010;152A:1688-1694.
59. Andersson O, Reissmann E, Jornvall H, *et al.* Synergistic interaction between Gdf1 and Nodal during anterior axis development. *Dev Biol* 2006;293:370-381.
60. Mo R, Freer AM, Zinyk DL, *et al.* Specific and redundant functions of Gli2 and Gli3 zinc finger genes in skeletal patterning and development. *Development* 1997;124:113-123.
61. Huang X, Goudy SL, Ketova T, *et al.* Gli3-deficient mice exhibit cleft palate associated with abnormal tongue development. *Dev Dyn* 2008;237:3079-3087.
62. Peyrard-Janvid M, Leslie EJ, Kousa YA, *et al.* Dominant mutations in GRHL3 cause Van der Woude Syndrome and disrupt oral periderm development. *Am J Hum Genet* 2014;94:23-32.
63. Shi M, Christensen K, Weinberg CR, *et al.* Orofacial cleft risk is increased with maternal smoking and specific detoxification-gene variants. *Am J Hum Genet* 2007;80:76-90.
64. Allanson JE, Gemmill RM, Hecht BK, *et al.* Deletion mapping of the beta-glucuronidase gene. *Am J Med Genet* 1988;29:517-522.
65. Gendron-Maguire M, Mallo M, Zhang M, *et al.* Hoxa-2 mutant mice exhibit homeotic transformation of skeletal elements derived from cranial neural crest. *Cell* 1993;75:1317-1331.
66. Barrow JR, Capecchi MR. Compensatory defects associated with mutations in Hoxa1 restore normal palatogenesis to Hoxa2 mutants. *Development* 1999;126:5011-5026
67. Moon H, Song J, Shin JO, *et al.* Intestinal cell kinase, a protein associated with endocrine-cerebro-osteodysplasia syndrome, is a key regulator of cilia length and Hedgehog signaling. *Proc Natl Acad Sci U S A* 2014;111:8541-8546.
68. Friedland-Little JM, Hoffmann AD, Ocbina PJ, *et al.* A novel murine allele of Intraflagellar Transport Protein 172 causes a syndrome including VACTERL-like features with hydrocephalus. *Hum Mol Genet* 2011;20:3725-3737.
69. Sohaskey ML, Yu J, Diaz MA, *et al.* JAWS coordinates chondrogenesis and synovial joint positioning. *Development* 2008;135:2215-2220.
70. Ingraham CR, Kinoshita A, Kondo S, *et al.* Abnormal skin, limb and craniofacial morphogenesis in mice deficient for interferon regulatory factor 6 (Irf6). *Nat Genet* 2006;38:1335-1340.
71. Richardson RJ, Dixon J, Malhotra S, *et al.* Irf6 is a key determinant of the keratinocyte proliferation-differentiation switch. *Nat Genet* 2006;38:1329-1334.
72. Vieira AR, Avila JR, Daack-Hirsch S, *et al.* Medical sequencing of candidate genes for nonsyndromic cleft lip and palate. *PLoS Genet* 2005;1:e64.
73. Jiang R, Lan Y, Chapman HD, *et al.* Defects in limb, craniofacial, and thymic development in Jagged2 mutant mice. *Genes Dev* 1998;12:1046-1057.
74. Zaritsky JJ, Eckman DM, Wellman GC, *et al.* Targeted disruption of Kir2.1 and Kir2.2 genes reveals the essential role of the inwardly rectifying K(+) current in K(+)-mediated vasodilation. *Circ Res* 2000;87:160-166.
75. Zhao Y, Guo YJ, Tomac AC, *et al.* Isolated cleft palate in mice with a targeted mutation of the LIM homeobox gene lhx8. *Proc Natl Acad Sci U S A* 1999;96:15002-15006.
76. Alzahrani F, Al Hazzaa SA, Tayeb H, *et al.* LOXL3, encoding lysyl oxidase-like 3, is mutated in a family with autosomal recessive Stickler syndrome. *Hum Genet* 2015;134:451-453.
77. Wheway G, Abdelhamed Z, Natarajan S, *et al.* Aberrant Wnt signalling and cellular over-proliferation in a novel mouse model of Meckel-Gruber syndrome. *Dev Biol* 2013;377:55-66.
78. Jugessur A, Shi M, Gjessing HK, *et al.* Genetic determinants of facial clefting: analysis of 357 candidate genes using two national cleft studies from Scandinavia. *PLoS One* 2009;4:e5385.
79. Letra A, da Silva RA, Menezes R, *et al.* Studies with MMP9 gene promoter polymorphism and nonsyndromic cleft lip and palate. *Am J Med Genet A* 2007;143A:89-91.
80. Satokata I, Maas R. Msx1 deficient mice exhibit cleft palate and abnormalities of craniofacial and tooth development. *Nat Genet* 1994;6:348-356.
81. Zhang Z, Song Y, Zhao X, *et al.* Rescue of cleft palate in Msx1-deficient mice by transgenic Bmp4 reveals a network of BMP and Shh signaling in the regulation of mammalian palatogenesis. *Development* 2002;129:4135-4146.
82. Lallemand Y, Moreau J, Cloment CS, *et al.* Generation and characterization of a tamoxifen inducible Msx1(CreERT2) knock-in allele. *Genesis* 2013;51:110-119.
83. Winograd J, Reilly MP, Roe R, *et al.* Perinatal lethality and multiple craniofacial malformations in MSX2 transgenic mice. *Hum Mol Genet* 1997;6:369-379.
84. Murthy J, Gurramkonda VB, Lakkakula BV. Significant association of MTHFD1 1958G>A single nucleotide polymorphism with nonsyndromic cleft lip and palate in Indian population. *Med Oral Patol Oral Cir Bucal* 2014;19:e616-621.
85. Martinelli M, Di Stazio M, Scapoli L, *et al.* Cleft lip with or without cleft palate: implication of the heavy chain of non-muscle myosin IIA. *J Med Genet* 2007;44:387-392.
86. Song T, Wu D, Wang Y, *et al.* Association of NAT1 and NAT2 genes with nonsyndromic cleft lip and palate. *Mol Med Rep* 2013;8:211-216.
87. Erickson RP, Cao W, Acuna DK, *et al.* Confirmation of the role of N-acetyltransferase 2 in teratogen-induced cleft palate using transgenics and knockouts. *Mol Reprod Dev* 2008;75:1071-1076.
88. Song T, Shi J, Guo Q, *et al.* Association between NOGGIN and SPRY2 polymorphisms and nonsyndromic cleft lip with or without cleft palate. *Am J Med Genet A* 2015;167A:137-141.
89. Shaw GM, Iovannisci DM, Yang W, *et al.* Endothelial nitric oxide synthase (NOS3) genetic variants, maternal smoking, vitamin use, and risk of human orofacial clefts. *Am J Epidemiol* 2005;162:1207-1214.
90. Leslie EJ, Taub MA, Liu H, *et al.* Identification of functional variants for cleft lip with or without cleft palate in or near PAX7, FGFR2, and NOG by targeted sequencing of GWAS loci. *Am J Hum Genet* 2015;96:397-411.
91. Ferrante MI, Zullo A, Barra A, *et al.* Oral-facial-digital type I protein is required for primary cilia formation and left-right axis specification. *Nat Genet* 2006;38:112-117.
92. Hyon C, Marlin S, Chantot-Bastaraud S, *et al.* A new 17p13.3 microduplication including the PAFAH1B1 and YWHAE genes resulting from an unbalanced X;17 translocation. *Eur J Med Genet* 2011;54:287-291.
93. Choi SJ, Marazita ML, Hart PS, *et al.* The PDGF-C regulatory region SNP rs28999109 decreases promoter transcriptional activity and is associated with CL/P. *Eur J Hum Genet* 2009;17:774-784.
94. Ding H, Wu X, Bostrom H, *et al.* A specific requirement for PDGF-C in palate formation and PDGFR-alpha signaling. *Nat Genet* 2004;36:1111-1116.
95. Rattanasopha S, Tongkobpetch S, Srichomthong C, *et al.* PDGFRa mutations in humans with isolated cleft palate. *Eur J Hum Genet* 2012;20:1058-1062.
96. Morrison-Graham K, Schatteman GC, Bork T, *et al.* A PDGF receptor mutation in the mouse (Patch) perturbs the development of a non-neuronal subset of neural crest-derived cells. *Development* 1992;115:133-142.
97. Tallquist MD, Soriano P. Cell autonomous requirement for PDGFRalpha in populations of cranial and cardiac neural crest cells. *Development* 2003;130:507-518.
98. Lanctot C, Moreau A, Chamberland M, *et al.* Hindlimb patterning and mandible development require the Ptx1 gene. *Development* 1999;126:1805-1810.
99. Szeto DP, Rodriguez-Esteban C, Ryan AK, *et al.* Role of the Bicoid-related homeodomain factor Pitx1 in specifying hindlimb morphogenesis and pituitary development. *Genes Dev* 1999;13:484-494.
100. Barrott JJ, Cash GM, Smith AP, *et al.* Deletion of mouse Porcn blocks Wnt ligand secretion and reveals an ectodermal etiology of human focal dermal hypoplasia/Goltz syndrome. *Proc Natl Acad Sci U S A* 2011;108:12752-12757.
101. Martin JF, Bradley A, Olson EN. The paired-like homeo box gene MHox is required for early events of skeletogenesis in multiple lineages. *Genes Dev* 1995;9:1237-1249.
102. ten Berge D, Brouwer A, Korving J, *et al.* Prx1 and Prx2 in skeletogenesis: roles in the craniofacial region, inner ear and limbs. *Development* 1998;125:3831-3842.
103. Lu MF, Cheng HT, Kern MJ, *et al.* prx-1 functions cooperatively with another paired-related homeobox gene, prx-2, to maintain cell fates within the craniofacial mesenchyme. *Development* 1999;126:495-504.
104. Metzis V, Courtney AD, Kerr MC, *et al.* Patched1 is required in neural crest cells for the prevention of orofacial clefts. *Hum Mol Genet* 2013;22:5026-5035.
105. Chenevix-Trench G, Jones K, Green AC, *et al.* Cleft lip with or without cleft palate: associations with transforming growth factor alpha and retinoic acid receptor loci. *Am J Hum Genet* 1992;51:1377-1385.
106. Mann MB, Hodges CA, Barnes E, *et al.* Defective sister-chromatid cohesion, aneuploidy and cancer predisposition in a mouse model of type II Rothmund-Thomson syndrome. *Hum Mol Genet* 2005;14:813-825.

107. Schwabe GC, Trepczik B, Suring K, *et al.* Ror2 knockout mouse as a model for the developmental pathology of autosomal recessive Robinow syndrome. *Dev Dyn* 2004;229:400-410.
108. Aberg T, Cavender A, Gaikwad JS, *et al.* Phenotypic changes in dentition of Runx2 homozygote-null mutant mice. *J Histochem Cytochem* 2004;52:131-139
109. Watanabe A, Akita S, Tin NT, *et al.* A mutation in RYK is a genetic factor for nonsyndromic cleft lip and palate. *Cleft Palate Craniofac J* 2006;43:310-316.
110. Halford MM, Armes J, Buchert M, *et al.* Ryk-deficient mice exhibit craniofacial defects associated with perturbed Eph receptor crosstalk. *Nat Genet* 2000;25:414-418.
111. Dobreva G, Chahrour M, Dautzenberg M, *et al.* SATB2 is a multifunctional determinant of craniofacial patterning and osteoblast differentiation. *Cell* 2006;125:971-986.
112. Britanova O, Depew MJ, Schwark M, *et al.* Satb2 haploinsufficiency phenocopies 2q32-q33 deletions, whereas loss suggests a fundamental role in the coordination of jaw development. *Am J Hum Genet* 2006;79:668-678.
113. Adly N, Alhashem A, Ammari A, *et al.* Ciliary genes TBC1D32/C6orf170 and SCLT1 are mutated in patients with OFD type IX. *Hum Mutat* 2014;35:36-40.
114. Huang X, Litingtung Y, Chiang C. Ectopic sonic hedgehog signaling impairs telencephalic dorsal midline development: implication for human holoprosencephaly. *Hum Mol Genet* 2007;16:1454-1468.
115. van Hasselt PM, Ferdinandusse S, Monroe GR, *et al.* Monocarboxylate transporter 1 deficiency and ketone utilization. *N Engl J Med* 2014;371:1900-1907.
116. Parada C, Li J, Iwata J, *et al.* CTGF mediates Smad-dependent transforming growth factor beta signaling to regulate mesenchymal cell proliferation during palate development. *Mol Cell Biol* 2013;33:3482-3493.
117. Rainger J, van Beusekom E, Ramsay JK, *et al.* Loss of the BMP antagonist, SMOC-1, causes Ophthalmo-acromelic (Waardenburg Anophthalmia) syndrome in humans and mice. *PLoS Genet* 2011;7:e1002114.
118. Viljoen DL, Smart R. Split-foot anomaly, microphthalmia, cleft-lip and cleft-palate, and mental retardation associated with a chromosome 6;13 translocation. *Clin Dysmorphol* 1993;2:274-277.
119. Bi W, Huang W, Whitworth DJ, *et al.* Haploinsufficiency of Sox9 results in defective cartilage primordia and premature skeletal mineralization. *Proc Natl Acad Sci U S A* 2001;98:6698-6703
120. Mori-Akiyama Y, Akiyama H, Rowitch DH, *et al.* Sox9 is required for determination of the chondrogenic cell lineage in the cranial neural crest. *Proc Natl Acad Sci U S A* 2003;100:9360-9365.
121. Welsh IC, Hagge-Greenberg A, O'Brien TP. A dosage-dependent role for Spry2 in growth and patterning during palate development. *Mech Dev* 2007;124:746-761.
122. Matsumura K, Taketomi T, Yoshizaki K, *et al.* Sprouty2 controls proliferation of palate mesenchymal cells via fibroblast growth factor signaling. *Biochem Biophys Res Commun* 2010;404:1076-1082.
123. Gohring I, Tagariello A, Endele S, *et al.* Disruption of ST5 is associated with mental retardation and multiple congenital anomalies. *J Med Genet* 2010;47:91-98.
124. Alkuraya FS, Saadi I, Lund JJ, *et al.* SUMO1 haploinsufficiency leads to cleft lip and palate. *Science* 2006;313:1751
125. Jerome LA, Papaioannou VE. DiGeorge syndrome phenotype in mice mutant for the T-box gene, Tbx1. *Nat Genet* 2001;27:286-291.
126. Funato N, Nakamura M, Richardson JA, *et al.* Tbx1 regulates oral epithelial adhesion and palatal development. *Hum Mol Genet* 2012;21:2524-2537.
127. Bush JO, Lan Y, Jiang R. The cleft lip and palate defects in Dancer mutant mice result from gain of function of the Tbx10 gene. *Proc Natl Acad Sci U S A* 2004;101:7022-7027.
128. Pauws E, Hoshino A, Bentley L, *et al.* Tbx22null mice have a submucous cleft palate due to reduced palatal bone formation and also display ankyloglossia and choanal atresia phenotypes. *Hum Mol Genet* 2009;18:4171-4179.
129. Martinelli M, Scapoli L, Palmieri A, *et al.* Study of four genes belonging to the folate pathway: transcobalamin 2 is involved in the onset of non-syndromic cleft lip with or without cleft palate. *Hum Mutat* 2006;27:294.
130. Dixon J, Brakebusch C, Fassler R, *et al.* Increased levels of apoptosis in the prefusion neural folds underlie the craniofacial disorder, Treacher Collins syndrome. *Hum Mol Genet* 2000;9:1473-1480.
131. Sang L, Miller JJ, Corbit KC, *et al.* Mapping the NPHP-JBTS-MKS protein network reveals ciliopathy disease genes and pathways. *Cell* 2011;145:513-528.
132. Brewer S, Feng W, Huang J, *et al.* Wnt1-Cre-mediated deletion of AP-2alpha causes multiple neural crest-related defects. *Dev Biol* 2004;267:135-152.
133. Sanford LP, Ormsby I, Gittenberger-de Groot AC, *et al.* TGFbeta2 knockout mice have multiple developmental defects that are non-overlapping with other TGFbeta knockout phenotypes. *Development* 1997;124:2659-2670.
134. Kaartinen V, Voncken JW, Shuler C, *et al.* Abnormal lung development and cleft palate in mice lacking TGF-beta 3 indicates defects of epithelial-mesenchymal interaction. *Nat Genet* 1995;11:415-421.
135. Proetzel G, Pawlowski SA, Wiles MV, *et al.* Transforming growth factor-beta 3 is required for secondary palate fusion. *Nat Genet* 1995;11:409-414.
136. Taya Y, O'Kane S, Ferguson MW. Pathogenesis of cleft palate in TGF-beta3 knockout mice. *Development* 1999;126:3869-3879.
137. Dudas M, Kim J, Li WY, *et al.* Epithelial and ectomesenchymal role of the type I TGF-beta receptor ALK5 during facial morphogenesis and palatal fusion. *Dev Biol* 2006;296:298-314.
138. Li WY, Dudas M, Kaartinen V. Signaling through Tgf-beta type I receptor Alk5 is required for upper lip fusion. *Mech Dev* 2008;125:874-882.
139. Ito Y, Yeo JY, Chytil A, *et al.* Conditional inactivation of Tgfbr2 in cranial neural crest causes cleft palate and calvaria defects. *Development* 2003;130:5269-5280.
140. Xu X, Han J, Ito Y, *et al.* Cell autonomous requirement for Tgfbr2 in the disappearance of medial edge epithelium during palatal fusion. *Dev Biol* 2006;297:238-248.
141. Yang A, Schweitzer R, Sun D, *et al.* p63 is essential for regenerative proliferation in limb, craniofacial and epithelial development. *Nature* 1999;398:714-718.
142. Blanc I, Bach A, Lallemand Y, *et al.* A new mouse limb mutation identifies a Twist allele that requires interacting loci on chromosome 4 for its phenotypic expression. *Mamm Genome* 2003;14:797-804.
143. Pizzuti A, Novelli G, Ratti A, *et al.* UFD1L, a developmentally expressed ubiquitination gene, is deleted in CATCH 22 syndrome. *Hum Mol Genet* 1997;6:259-265.
144. Bertuzzi S, Hindges R, Mui SH, *et al.* The homeodomain protein vax1 is required for axon guidance and major tract formation in the developing forebrain. *Genes Dev* 1999;13:3092-3105
145. Ashe A, Butterfield NC, Town L, *et al.* Mutations in mouse Ift144 model the craniofacial, limb and rib defects in skeletal ciliopathies. *Hum Mol Genet* 2012;21:1808-1823.
146. Nimura K, Ura K, Shiratori H, *et al.* A histone H3 lysine 36 trimethyltransferase links Nkx2-5 to Wolf-Hirschhorn syndrome. *Nature* 2009;460:287-291.
147. He F, Xiong W, Yu X, *et al.* Wnt5a regulates directional cell migration and cell proliferation via Ror2-mediated noncanonical pathway in mammalian palate development. *Development* 2008;135:3871-3879.
148. Yamaguchi TP, Bradley A, McMahon AP, *et al.* A Wnt5a pathway underlies outgrowth of multiple structures in the vertebrate embryo. *Development* 1999;126:1211-1223.
149. Jiang Z, Zhu L, Hu L, *et al.* Zic3 is required in the extra-cardiac perinodal region of the lateral plate mesoderm for left-right patterning and heart development. *Hum Mol Genet* 2013;22:879-889.
